# Personalizing esketamine treatment in TRD and TRBD: the role of mentalization, cognitive rigidity, psychache, and suicidality

**DOI:** 10.3389/fpsyt.2025.1736114

**Published:** 2026-01-22

**Authors:** Miriam Olivola, Filippo Mazzoni, Barbara Tarantino, Alessandro Guffanti, Matteo Marcatili, Monica Macellaro, Nicolaja Girone, Vassilis Martiadis, Fabiola Raffone, Tiziano Prodi, Natascia Brondino, Giovanni Martinotti, Massimo Clerici, Roberta Anniverno, Bernardo Dell’Osso

**Affiliations:** 1 Department of Brain and Behavioural Sciences, University of Pavia, Pavia, Italy; 2 Department of Mental Health and Addiction Services, Azienda Socio Sanitaria Territoriale (ASST) Fatebenefratelli-Sacco, Milan, Italy; 3 Department of Mental Health, Fondazione Istituto di Ricovero e Cura a Carattere Scientifico (IRCSS) San Gerardo dei Tintori, Monza, Italy; 4 Department of Mental Health, University of Milan, Milan, Italy; 5 Department of Biomedical and Clinical Sciences “Luigi Sacco”, University of Milan, Milan, Italy; 6 Department of mental health , Azienda Sanitaria Locale (ASL) Naples 1 Centre, Naples, Italy; 7 Department of Neuroscience, Imaging and Clinical Sciences, “G. D’Annunzio” University, Chieti, Italy; 8 School of Medicine and Surgery, University of Milano-Bicocca, Milan, Italy; 9 ”Aldo Ravelli” Center for Nanotechnology and Neurostimulation, University of Milan, Milan, Italy; 10 Department of Psychiatry and Behavioral Sciences, Stanford University, Stanford, CA, United States; 11 Centro per lo studio dei meccanismi molecolari alla base delle patologie neuro-psico-geriatriche”, University of Milan, Milan, Italy

**Keywords:** cognitive rigidity, esketamine, major depressive disorder, mentalization, psychache, treatment-resistant depression

## Abstract

**Introduction:**

Treatment-Resistant Depression (TRD) remains a major challenge in the management of Major Depressive Disorder (MDD). Esketamine, the S-enantiomer of ketamine and a glutamatergic modulator, was approved by the FDA and EMA for TRD in 2019. Beyond its rapid antidepressant effects, esketamine may enhance neuroplasticity, facilitating the reconnection with emotional and cognitive processes, improving mentalization and social cognition, and promoting resilience.

**Objective:**

This prospective multicenter observational study aimed to evaluate esketamine’s therapeutic impact on both depressive symptoms and key psychological factors—including mentalization, psychache, social cognition, suicidality, and cognitive-emotional rigidity—that could predict treatment response and enable a more personalized approach to TRD and TRBD management.

**Methods:**

Thirty-six treatment-resistant depressive episode patients, including TRD and TRB, treated with esketamine were assessed over a six-month follow-up period using psychometric measures of depression severity, suicidality, mentalization, social cognition, psychache, and cognitive-emotional rigidity.

**Results:**

A significant improvement in depressive symptoms was observed, as indicated by a reduction in Montgomery-Åsberg Depression Rating Scale (MADRS) scores over time. Moreover, improvement was observed in different key psychological domains, such as mentalization, psychache, social cognition, suicidality, and cognitive-emotional rigidity. By six months, 69% of patients achieved remission, confirming a robust and sustained therapeutic response.

**Conclusions:**

These findings highlight the importance of a personalized treatment approach in treatment- resistant depressive episode patients. Esketamine may be particularly beneficial in reducing cognitive rigidity, improving mentalization, and breaking the cognitive inflexibility that contributes to sustained negative depressive thinking patterns. Further research is needed to refine patient stratification and optimize treatment strategies for individuals with treatment-resistant depressive episode patients.

## 1 Introduction

Treatment-Resistant Depression (TRD) represents a major clinical challenge in the management of Major Depressive Disorder (MDD). It is commonly defined by the failure to achieve an adequate therapeutic response following at least two distinct antidepressant treatments, each administered at appropriate dosages and for a sufficient duration (1). Although conventional antidepressants typically require several weeks to exert measurable effects, early symptomatic improvement may emerge within the first two weeks of treatment and has been consistently associated with a higher likelihood of achieving remission (2, 3). Conversely, a suboptimal early response—often defined as less than a 20% reduction in symptom severity after two to four weeks—frequently prompts treatment modifications, such as dose escalation or switching strategies (4). Despite these interventions, approximately 20% of patients with depression remain unresponsive to standard pharmacological approaches (5).

To date, electroconvulsive therapy (ECT) remains the gold standard for the treatment of TRD, particularly in the presence of severe, life-threatening symptoms, high suicidal risk, or psychotic features (6). However, its use is often limited by reduced accessibility, logistical constraints, and persistent social stigma (7). In response to these limitations, alternative therapeutic strategies have been developed, including neuromodulation techniques such as repetitive transcranial magnetic stimulation (rTMS) and novel pharmacological approaches, most notably intranasal esketamine (8, 9).

Esketamine, the S-enantiomer of ketamine, has demonstrated significant efficacy in the treatment of TRD, when administered in combination with SSRIs or SNRIs (10). Following positive results from multiple randomized clinical trials, esketamine has received regulatory approval from both the U.S. Food and Drug Administration (FDA) and the European Medicines Agency (EMA) for the treatment of TRD (11–14). Pharmacodynamically, esketamine acts as a non-competitive antagonist of the N-methyl-D- aspartate (NMDA) receptor, leading to modulation of glutamatergic neurotransmission (14, 15).

The tolerability and safety profile of intranasal esketamine have been extensively evaluated in both controlled trials and real-world clinical settings (14, 16, 17). The most frequently reported adverse effects include transient dissociative symptoms, such as alterations in body perception, depersonalization, and derealization (18), as well as mild and short-lived events including hypertension, nausea, dizziness, vertigo, sedation, hypoesthesia, and anxiety (18, 19). Notably, older adults appear to be more susceptible to dizziness and sedation (17).

Several neurobiological mechanisms have been proposed to account for the rapid antidepressant and antisuicidal effects of esketamine, although these pathways are not yet fully elucidated. Consistent evidence indicates a rapid reduction in suicidal ideation and depressive symptoms following treatment initiation (20), while full symptomatic remission may require up to three months of continued administration (14). One of the most widely supported mechanisms underlying esketamine’s clinical efficacy involves the enhancement of neuroplasticity, particularly through activation of the mammalian target of rapamycin (mTOR) and brain- derived neurotrophic factor (BDNF) signaling pathways. The enhancement of neuroplasticity may support the reorganization and reconnection of cognitive and emotional networks, thereby increasing psychological resilience and enabling patients to engage more effectively in psychotherapeutic and psychosocial interventions, with potential benefits for long-term functional recovery.

Recent narrative syntheses have emphasized the relevance of these strategies in moving beyond purely symptom-based paradigms toward mechanism-informed and personalized treatment models in TRD (21).

Moreover, significant improvements in anhedonic symptoms have been shown, supporting the view that esketamine acts on clinically relevant domains beyond core depressive severity. In line with these observations, REAL-ESK findings support the effectiveness of intranasal esketamine in patients with treatment-resistant bipolar depression (TRBD), with sustained clinical benefits over time, with a favorable tolerability profile and, notably, with a low risk of manic switch.

Despite its therapeutic potential, esketamine treatment also presents relevant limitations, including logistical challenges related to in-clinic administration, prescribing and monitoring requirements, and the persistence of social stigma surrounding both the drug and psychiatric interventions more broadly (7, 14, 16). Addressing these barriers is essential to ensure broader accessibility, adherence, and integration into routine clinical care. Beyond symptom severity, dimensions such as psychache, mentalization/social cognition, and cognitive-emotional rigidity may sustain distress and suicidal risk and may be clinically relevant targets of change. We therefore examined longitudinal changes in these domains over six months of intranasal esketamine treatment in patients with TRD/TRD-B.

## 2 Materials and methods

### 2.1 Participants and study design

We conducted a prospective, multicenter observational study at the Department of Mental Health and Addiction Services of Pavia, the IRCCS San Gerardo in Monza and the ‘G. d’Annunzio’ University of Chieti. The study aimed to evaluate both primary and secondary outcomes to assess the therapeutic effects of esketamine. The primary outcome was depressive symptom severity, while secondary outcomes included suicidality, psychache, mentalization, social cognition, emotional rigidity, and dissociative symptoms. The primary outcome, the severity of depressive symptoms, was measured using the Montgomery-Åsberg Depression Rating Scale (MADRS) (22). This scale was selected due to its established reliability and validity in assessing depression severity in patients with TRD and TRD-B. Furthermore, the overtime change in the Columbia-Suicide Severity Rating Scale (C-SSRS) (23), the Psychache Scale (PSA) (24), the Reading the Mind in the Eyes Test (RMET) (25), the Reflective Functioning Questionnaire (RFQ-8) (26), and the Resistance to Change Scale (RTC) (27) were used to assess secondary outcomes.

We also analyzed the RTC subscales—cognitive, emotional, and organizational resistance—to provide a more granular understanding of patients’ rigidity profiles.

The present research aimed secondarily to evaluate the tolerability of esketamine by assessing dissociative symptoms using the Dissociative Experience Scale-II (DES-II) (28).

Inclusion criteria included age 18–80 years, meeting criteria for TRD as defined by McIntyre et al. (1), and be experiencing a moderate-to-severe depressive episode in Major Depressive Disorder or Bipolar Disorder, with a MADRS score of ≥20. Of the 36 participants included in the study, only two had previously been diagnosed with bipolar disorder. Notably, neither had experienced hypomanic or manic episodes for over 10 years prior to enrolment. Their clinical presentation over the past decade was characterised exclusively by recurrent depressive episodes, consistent with a predominantly depressive polarity over a long period of time. Furthermore, participants needed to possess sufficient cognitive capacity to understand the study procedures and were evaluated with the Mini-Mental State Examination (MMSE) considering a score of 24 or more, to confirm capacity to provide informed consent. Exclusion criteria included hypersensitivity to ketamine or any of its excipients, a history of aneurysmal vascular disease, intracerebral hemorrhage, or cardiovascular events in the preceding six weeks. Additional exclusion criteria comprised any condition where an increase in blood pressure or intracranial pressure posed a significant risk, untreated hyperthyroidism, a history of psychotic disorders, or active substance use disorder. Participants were enrolled consecutively among eligible patients. Participants were enrolled between June 2021 and June 2024, with a follow-up period lasting six months for each patient. Informed consent was obtained before the administration of any study-related questionnaires, following a comprehensive explanation of the study’s objectives and procedures. Sociodemographic and clinical data were collected at baseline, included information on the treatment history, current pharmacotherapy, bipolar diathesis, current and previous nonpharmacological therapies, family history and personality disorder, age at first MDE, number of previous antidepressants, number of previous antipsychotics, current MDE, duration of MDE, number of previous SNRIs, number of previous MDE, number of previous MDE. Treatment resistance was verified through a structured review of each patient’s antidepressant history using a checklist based on the Antidepressant Treatment History Form – Short Form (ATHF - SF) (29), evaluating dose adequacy, treatment duration, adherence, and therapeutic class. Patients whose previous treatments did not meet adequacy criteria were not eligible for inclusion. The first psychometric assessment (T0) was conducted before treatment initiation. Subsequent assessments were performed at one week (T1), one month (T2), two months (T3), three months (T4), and six months (T5) after treatment initiation. Psychometric evaluations were always conducted before the administration of esketamine.

### 2.2 Intervention

Esketamine was administered as a nasal spray under clinical supervision following established guidelines. The treatment schedule followed a flexible dosing regimen, starting with an initial dose of 56 mg for the first session, followed by 56 mg or 84 mg per session based on clinical response and tolerability. Induction phase (Weeks 1–4): esketamine was administered twice weekly. Optimization phase (Weeks 5–8): administration frequency was adjusted to once weekly based on clinical improvement. Maintenance phase (Weeks 9–24): esketamine was administered every one to two weeks, depending on individual patient response and clinician judgment. Each session lasted approximately 2 hours, during which patients were monitored for potential adverse effects, including transient increases in blood pressure, dissociative symptoms, and sedation. Blood pressure was measured before and 40 minutes after administration, and patients remained under medical supervision until clinically stable. This protocol was designed to ensure both safety and efficacy, allowing for personalized adjustments in dosage and frequency based on patient response. The dosing schedule and administration procedure followed international regulatory guidelines and expert recommendations, including the FDA Prescribing Information (SPRAVATO^®^, 2019), the EMA EPAR (2019), and published consensus and clinical evidence (10, 13, 20). All study procedures were conducted in accordance with Good Clinical Practice (GCP) guidelines and the principles of the Declaration of Helsinki. Ethical approval for the study was granted by the Pavia Ethics Committee during its session on August 27, 2021 (Opinion No. 84157/21), with a subsequent amendment issued under No. 0102231/21.

### 2.3 Statistical analysis

All statistical analyses were performed in R (v.4.3.1). Descriptive statistics were first computed to provide an overview of the sample: continuous variables are reported as mean ± standard deviation (SD), while categorical variables are summarized as absolute frequencies and percentages.

Given the relatively small sample size and the non-normal distribution of several outcomes, we relied on non- parametric methods. Longitudinal changes in psychometric measures (MADRS (22), DES-II (28), Psychache Scale (24), RFQ-8 (26), RMET (25), RTC (27), and C-SSRS (23) items were evaluated by comparing each follow-up visit (T1–T5) with baseline (T0). For continuous outcomes, we used the paired Wilcoxon signed- rank test; for categorical endpoints (response and remission rates based on MADRS), we applied McNemar’s test for paired proportions. To control for multiple testing, p-values were adjusted using the Benjamini– Hochberg procedure. Given the relatively small sample size and the non-normal distribution of several psychometric measures, non-parametric paired tests were selected to provide robust inference without relying on assumptions that may not hold in this dataset. Mixed-effects models were considered but deemed less appropriate in light of sample size and distributional characteristics.

Missing data were managed through complete-case analysis, as the overall amount of missingness was limited and randomly distributed across variables.

All tests were two-sided, with a significance threshold set at p < 0.05. Considering the large number of item- level comparisons across psychometric scales [particularly MADRS (22) and C-SSRS (23)], the interpretation of results was deliberately focused on findings that remained significant after multiple testing correction, in order to highlight the most robust and clinically meaningful effects.

Given the very small number of patients with treatment-resistant bipolar depression (TRBD) included in the sample (n = 2), and the observation that their baseline and longitudinal psychometric scores were largely overlapping with those of patients with unipolar TRD, no separate subgroup analyses were performed for TRBD.

## 3 Results

### 3.1 Sample characteristics

The final cohort comprised 36 individuals diagnosed with TRD and TRD-B. The mean age was 45.9 ± 18.0 years, and 15 participants (41.7%) were male. At baseline, the average duration of the current major depressive episode exceeded 17 months,. Most participants (86.1%) reported no history of hypomanic or manic episodes, suggesting limited evidence for bipolar diathesis.

**Figure 1:**
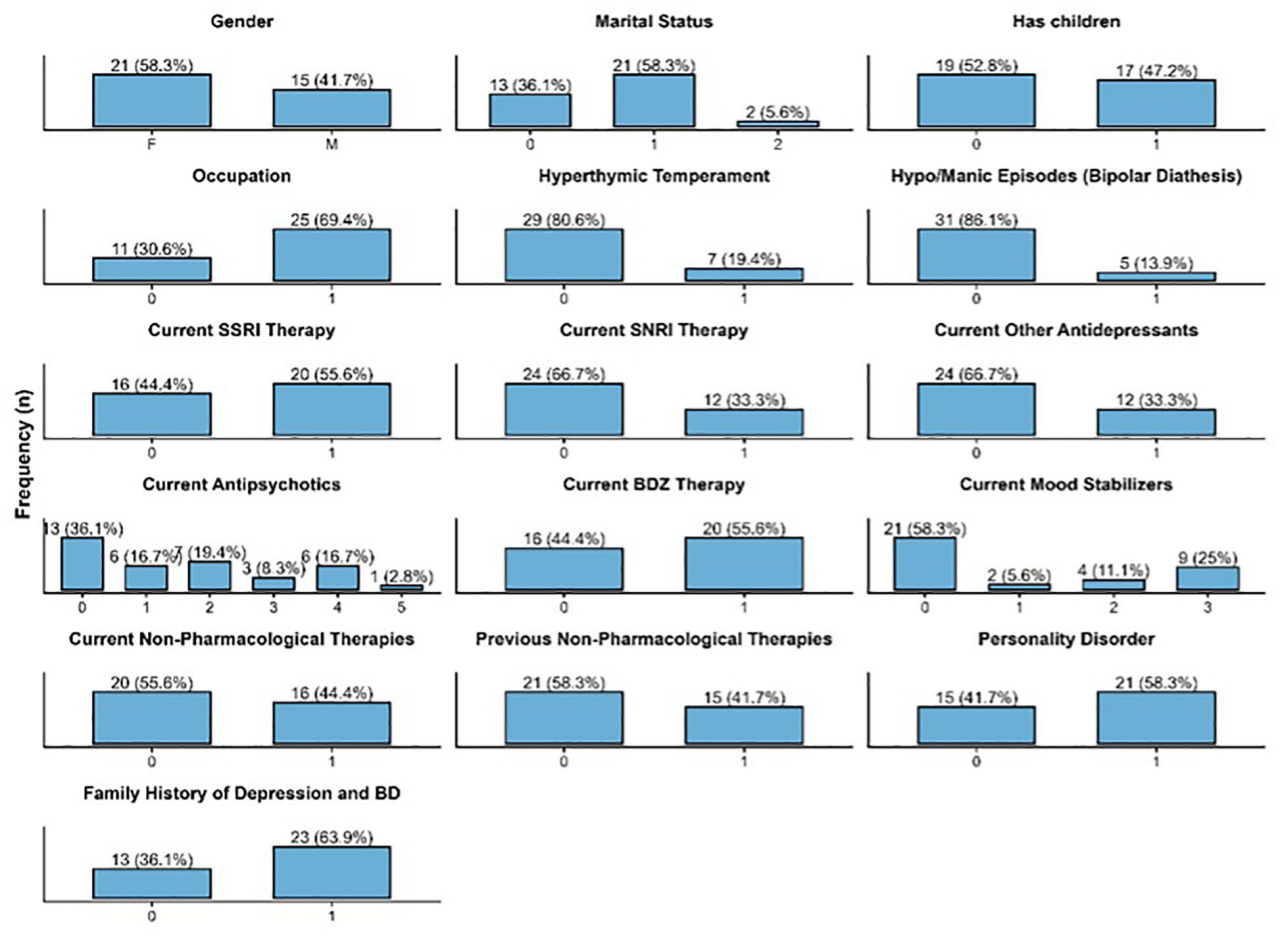
Distribution of sociodemographic and clinical characteristics in the study sample. Categorical variables are coded as follows: • Marital Status: 0 = Single, 1 = Married, 2 = Divorced/Separated. • Children: 0 = No children, 1 = ≥1 child. • Occupation: 0 = Unemployed, 1 = Employed. • Hyperthymic Temperament: 0 = Absent, 1 = Present. • Hypo/Manic Episodes Bipolar Diathesis: No = 0, Yes = 1. • Current SSRI/SNRI/Other Antidepressants/BZD:. 0 = Not in treatment, 1 = In treatment. • Current Antipsychotic Therapy (Brepiprazole = 1, Aripiprazole = 2, Olanzapine = 3, Quetiapine = 4, Amisulpride = 5, Cariprazine = 6). • Current Mood Stabilizer Therapy (Valproic Acid = 1, Lithium = 2, Gabapentin/Pregabalin = 3, No = 0). • Non-Pharmacological Therapies (No = 0, Psychotherapy = 1). • Previous Therapies (SSRI, SNRI, Antipsychotics, Other Antidepressants): Number of previous pharmacological trials in each class. • Personality Disorder: 0 = Absent, 1 = Present. • Family History of Depression and BD: 0 = No family history; 1 = Positive family history (depression and/or bipolar disorder).

Comorbidities were frequentwith a mean of 1.6 concurrent medical or psychiatric conditions, and more than half met criteria for a comorbid personality disorder. Regarding pharmacological treatments, 55.6% were receiving SSRIs, 33.3% SNRIs, and many were under polypharmacy regimens including antipsychotics or mood stabilizers. Sociodemographic and categorical clinical characteristics are summarized in **Figure 1**, while descriptive statistics for continuous variables are reported in **Table 1**. Five participants (13.9%) discontinued the study during follow-up: four within the first week and one after one month. Reported reasons were lack of perceived efficacy (n = 2), work-related difficulties (n = 2), and protocol violation due to acute cocaine intoxication (n = 1).

**Table 1:** Sociodemographic and clinical features.

Variable	N	Mean	Min	Max	SD
Age (years)	36	45.94	19	79	18.00
Age at First MDE	36	30.83	12	79	15.32
Number of Previous Antidepressants	36	1.08	0	5	1.13
Number of Previous Antipsychotics	36	1.28	0	6	1.37
Number of Comorbidities	36	1.64	0	5	1.33
Current MDE Duration	36	17.34	3	84	15.49
Number of Previous SNRIs	36	0.64	0	2	0.68
Number of Previous MDE	36	4.78	0	40	6.71
Number of suicidal attempts	36	0.72	0	3	0.97

### 3.2 Clinical response and remission

**Figure 2:**
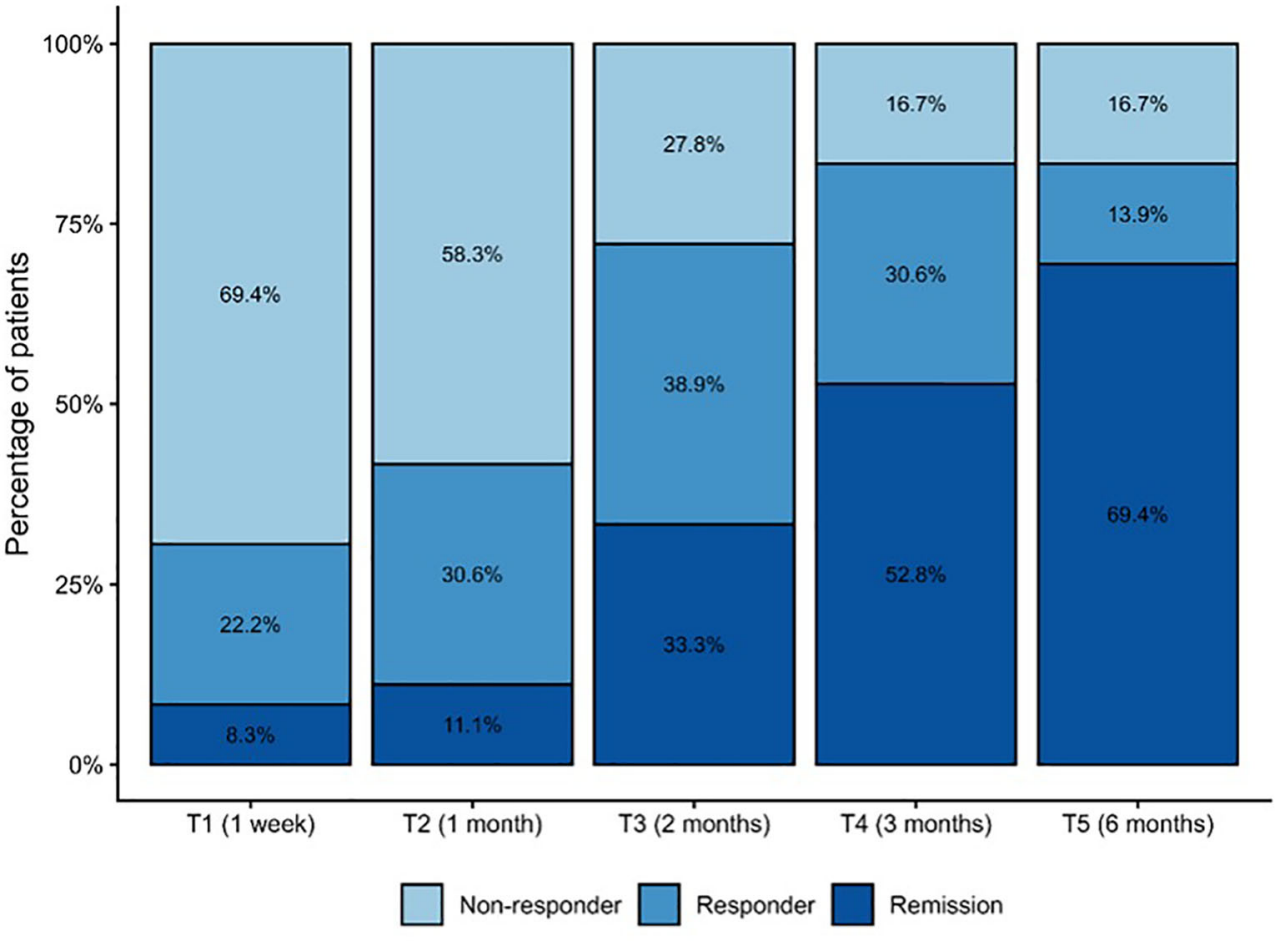
Clinical response and remission rates across timepoints.

Rates of clinical response and remission are presented in **Table 2** and **Figure 2**. At the first week of treatment (T1), most patients remained non-responders (69.4%), while 22.2% showed clinical response and 8.3% achieved remission. At one month (T2), the proportion of non-responders decreased to 58.3%, with 30.6% responders and 11.1% in remission. More marked improvements were observed at two months (T3), with 27.8% non-responders, 38.9% responders, and 33.3% in remission. By three months (T4), remission became the most common outcome (52.8%), while only 16.7% remained non-responders. At six months (T5), remission rates increased further to 69.4%, with 13.9% responders and 16.7% non-responders.

**Table 2:** Psychometric evaluation (mean ± SD at each timepoint).

Variable	T0	T1	T2	T3	T4	T5
Behavioral resistance (RTC-Behavioral)	3.89 (1.22)	3.71 (1.21)	3.71 (1.21)	3.71 (1.21)	3.71 (1.21)	3.71 (1.21)
Cognitive resistance (RTC-Cognitive)	4.27 (1.34)	3.68 (1.32)	4.20 (1.26)	3.56 (0.85)	3.24 (0.89)	3.60 (1.41)
Emotional resistance (RTC-Emotional)	4.95 (1.13)	4.31 (1.56)	4.89 (1.12)	3.81 (1.32)	2.73 (1.55)	2.62 (1.63)
Depressive symptoms (MADRS Total)	35.33 (8.30)	23.56 (10.93)	18.06 (8.21)	12.32 (6.46)	10.48 (7.87)	7.58 (4.41)
Mentalization (RFQ-8 Total)	3.29 (1.33)	2.95 (1.01)	3.05 (1.13)	2.65 (0.96)	2.63 (0.89)	2.36 (0.74)
Organizational_Resistance_Score (1-5)	3.94 (0.85)	3.83 (0.85)	3.83 (0.85)	3.83 (0.85)	3.83 (0.85)	3.73 (0.96)
PSA Total	49.17 (11.23)	40.56 (13.98)	34.94 (13.67)	28.97 (12.09)	21.84 (9.84)	16.77 (4.60)
RMET Total	NA	22.31 (9.60)	24.23 (6.50)	24.29 (7.02)	28.68 (5.84)	28.74 (5.56)
DES-II Total	0.17 (0.13)	0.17 (0.17)	0.15 (0.14)	0.14 (0.13)	0.11 (0.13)	0.08 (0.08)
C-SSRS Item 1	1.00 (0.00)	0.44 (0.50)	0.26 (0.44)	0.26 (0.44)	0.19 (0.40)	0.03 (0.18)
C-SSRS Item 2	0.50 (0.51)	0.19 (0.40)	0.13 (0.34)	0.13 (0.34)	0.19 (0.40)	0.03 (0.18)
C-SSRS Item 3	0.39 (0.49)	0.09 (0.30)	0.10 (0.30)	0.10 (0.30)	0.13 (0.34)	0.00 (0.00)
C-SSRS Item 4	0.31 (0.47)	0.12 (0.34)	0.10 (0.30)	0.16 (0.37)	0.10 (0.30)	0.03 (0.18)
C-SSRS Item 5	0.14 (0.35)	0.00 (0.00)	0.03 (0.18)	0.03 (0.18)	0.03 (0.18)	0.00 (0.00)
C-SSRS Item 6	2.44 (1.66)	1.34 (0.94)	1.00 (1.26)	0.94 (1.12)	0.42 (0.50)	0.23 (0.50)
C-SSRS Item 6.1	2.06 (1.47)	1.47 (1.27)	0.97 (1.17)	0.87 (1.09)	0.45 (0.51)	0.19 (0.40)
C-SSRS Item 6.2	1.67 (1.24)	1.22 (0.83)	0.87 (0.85)	0.90 (0.91)	0.48 (0.57)	0.26 (0.44)
C-SSRS Item 6.3	1.92 (1.42)	1.22 (1.10)	1.00 (1.32)	1.16 (1.42)	0.48 (0.68)	0.26 (0.51)
C-SSRS Item 6.4	1.31 (1.04)	1.22 (1.18)	0.77 (0.80)	0.77 (0.96)	0.55 (0.85)	0.16 (0.37)
C-SSRS Item 6.5	2.86 (1.93)	1.94 (1.88)	1.55 (1.80)	1.03 (1.60)	0.55 (0.77)	0.26 (0.51)
C-SSRS Item 7	0.25 (0.44)	0.28 (0.46)	0.10 (0.30)	0.16 (0.37)	0.13 (0.34)	0.03 (0.18)
C-SSRS Item 7.1	0.60 (0.95)	0.53 (0.90)	0.23 (0.68)	0.28 (0.75)	0.29 (0.74)	0.10 (0.40)
C-SSRS Item 7.2	0.33 (0.48)	0.19 (0.40)	0.16 (0.37)	0.16 (0.37)	0.23 (0.43)	0.10 (0.30)
C-SSRS Item 7.3	0.28 (0.61)	0.23 (0.62)	0.06 (0.25)	0.00 (0.00)	0.10 (0.40)	0.06 (0.36)
C-SSRS Item 7.4	0.42 (0.97)	0.29 (0.64)	0.23 (0.67)	0.29 (0.82)	0.29 (0.82)	0.19 (0.65)
C-SSRS Item 7.5	0.28 (0.45)	0.09 (0.30)	0.06 (0.25)	0.06 (0.25)	0.03 (0.18)	0.03 (0.18)
C-SSRS Item 7.6	0.11 (0.32)	0.06 (0.25)	0.03 (0.18)	0.06 (0.25)	0.03 (0.18)	0.03 (0.18)

MADRS, Montgomery–Åsberg Depression Rating Scale; C-SSRS, C-SSRS-Suicide Severity Rating Scale; DES-II, Dissociative Experience Scale-II; PSA, Psychache Scale; RMET, Reading the Mind in the Eyes Test; RFQ- 8, Reflective Functioning Questionnaire; RTC, Resistance to Change Scale.

### 3.3 Psychometric outcomes

**Figure 3:**
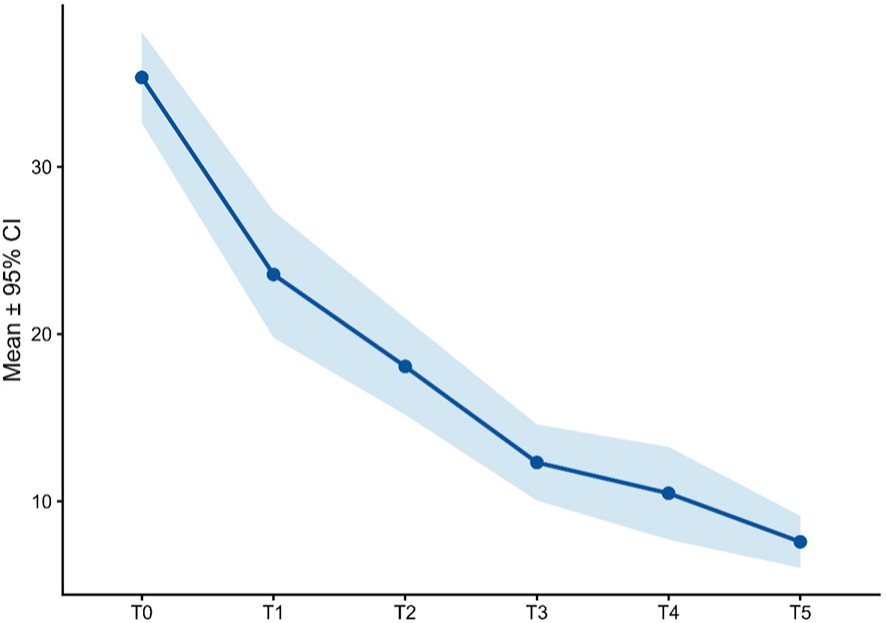
MADRS total score over time.

**Figure 4:**
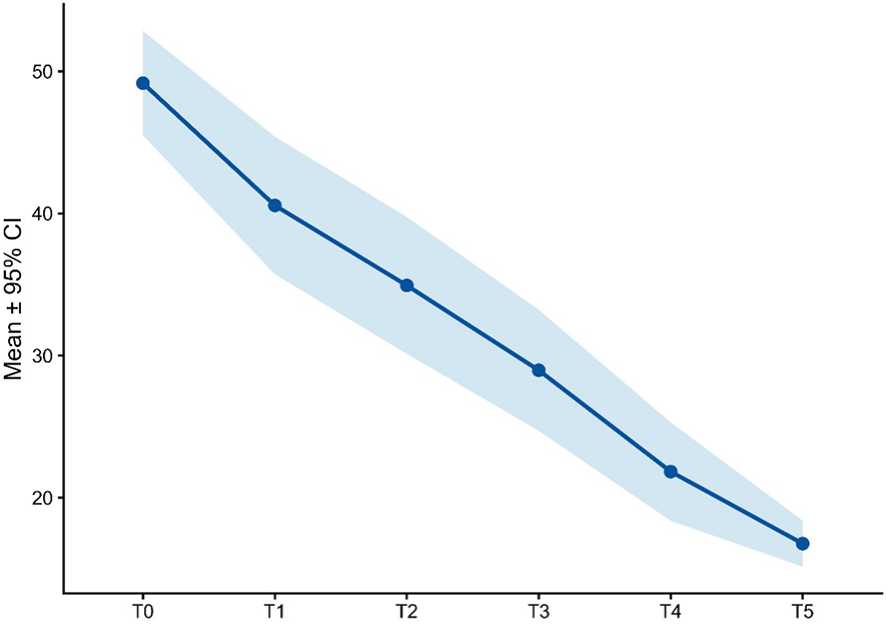
PSA score over time.

**Figure 5:**
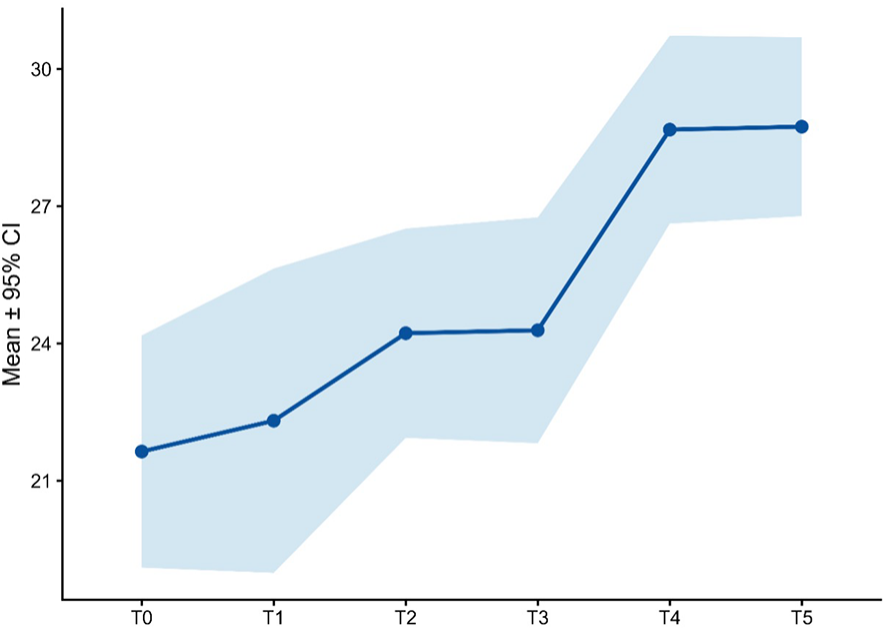
RMET score over time.

**Figure 6:**
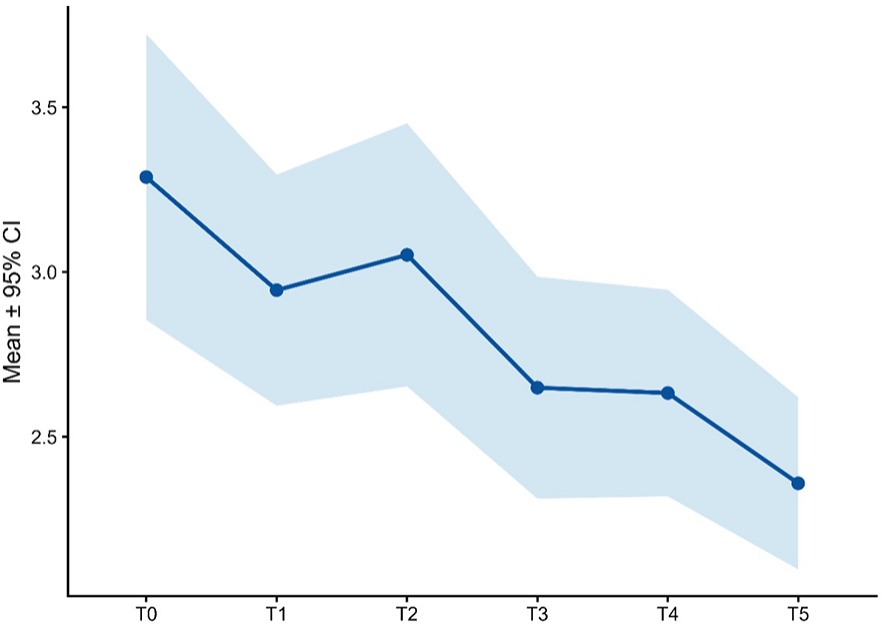
RFQ score over time.

**Figure 7:**
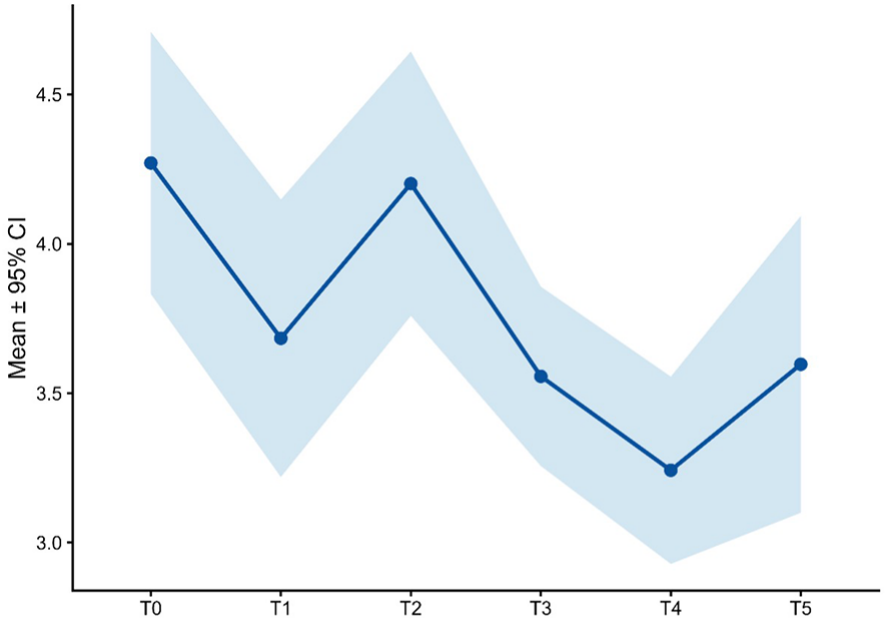
RTC Cognitive Resistance score over time.

**Figure 8:**
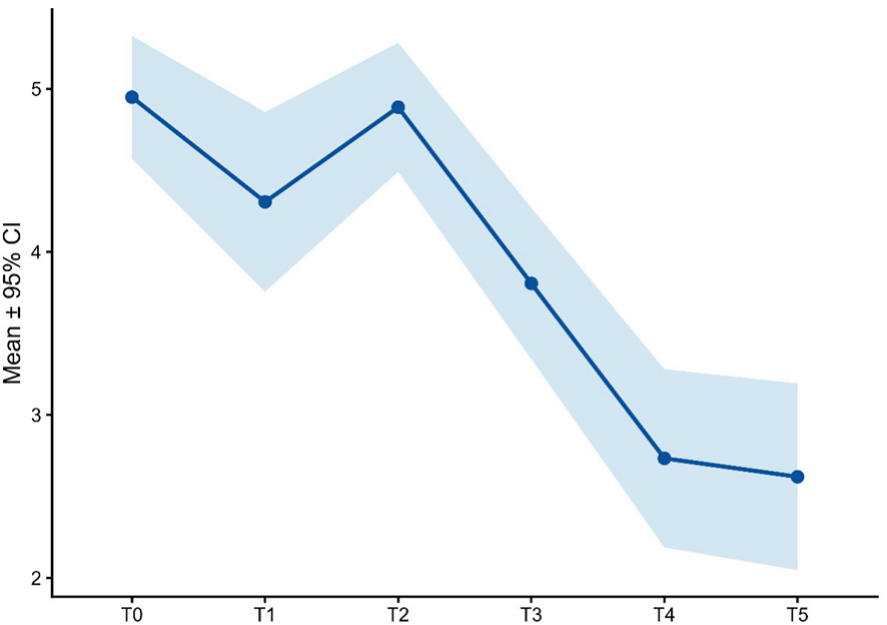
RTC Emotional Resistance score over time.

**Figure 9:**
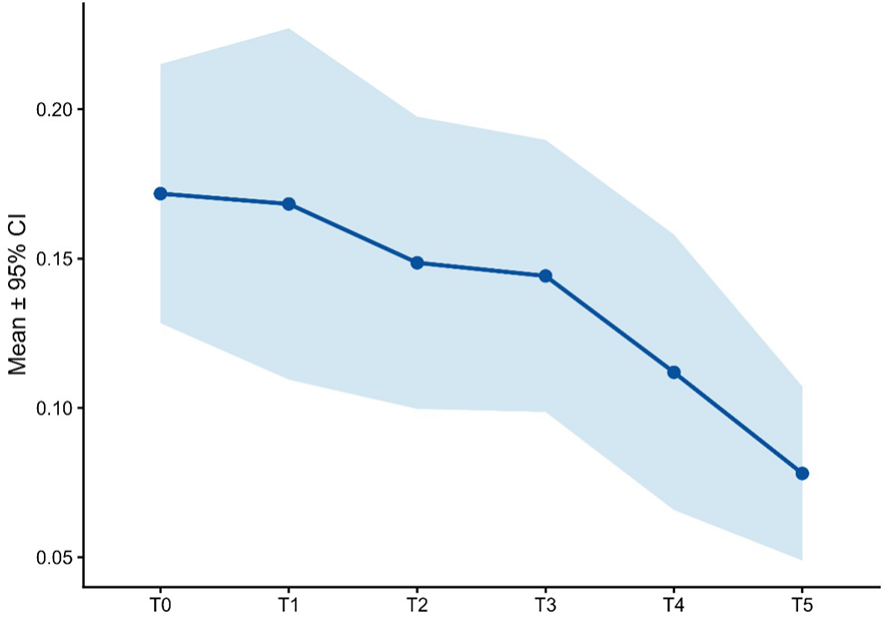
DES-II score over time.

Psychometric outcomes are summarized in **Table 2, Supplementary Tables S1-S2** and **Figures 3**–**9**. At baseline, the mean MADRS total score was 35.3 ± 8.3, consistent with severe depression. Marked reductions were already evident at T1 (23.6 ± 10.9) and consolidated at T2 (18.1 ± 8.2). Symptoms continued to improve at T3 (12.3 ± 6.5), T4 (10.5 ± 7.9), and T5 (7.6 ± 4.4). Wilcoxon signed-rank tests confirmed highly significant reductions in MADRS total scores compared with baseline at all follow-ups (all adjusted p < 0.001; **Supplementary Table S1**).

Together, these results indicate both global and domain-specific improvements in depressive symptomatology over the course of treatment (**Figure 3**).

Suicidality, assessed using the Columbia–Suicide Severity Rating Scale (C-SSRS; **Table 2** and **Supplementary Table S2**), was prominent at baseline, with elevated scores for wish to be dead (Item 1: mean = 1.00), non-specific active suicidal thoughts (Item 2: 0.50 ± 0.5), and suicidal behavior (Item 6: 2.44 ± 1.7). A marked reduction in suicidality was already evident at the first follow-up (T1), with further progressive declines observed through T5). Overall, these findings indicate a rapid, sustained, and statistically robust reduction in both suicidal ideation and behavior during esketamine treatment, with no evidence of early worsening at any timepoint.

Beyond depressive severity and suicidality, esketamine treatment was associated with convergent improvements across psychological pain, social-cognitive functioning, and flexibility-related domains.

Baseline psychache scores were high (49.2 ± 11.2). Mean scores declined progressively to 40.6 ± 14.0 at T1, 34.9 ± 13.7 at T2, and 29.0 ± 12.1 at T3. The reduction continued through T4 (21.8 ± 9.8) and T5 (16.8 ± 4.6). This trajectory suggests a marked alleviation of psychological pain in parallel with clinical improvement (**Figure 4**, **Table 2**).

Social-cognitive functioning, assessed through complementary self-report and performance-based measures, showed a gradual and coherent pattern of improvement over the course of treatment. Reflective functioning, measured by the RFQ-8, was impaired at baseline (mean = 3.29 ± 1.3) and improved progressively over time, with early reductions evident at T1 (2.95 ± 1.0), relative stabilization during mid-treatment (T2–T3; approximately 3.05–2.65), and a more pronounced improvement at the endpoint (T5: 2.36 ± 0.7; **Figure 6**).

In parallel, theory of mind performance, assessed using the Reading the Mind in the Eyes Test (RMET), increased steadily across follow-up assessments. Although no baseline RMET measure was available, mean scores rose from 22.3 ± 9.6 at T1 to 24.3 ± 6.5 at T2, reaching a peak of 28.7 ± 5.6 at T5 (**Figure 5**), suggesting an enhanced ability to decode complex emotional and mental states over time. While a contribution of test–retest learning effects cannot be entirely excluded due to repeated administration of the same instrument, the concordant improvement observed across both subjective and performance-based indices supports a meaningful enhancement of social-cognitive functioning during treatment.

Resistance to change, explored across cognitive, emotional, and behavioral domains, showed differentiated trajectories over the course of esketamine treatment (**Table 2**; **Figures 7**, **8**). Cognitive rigidity decreased from baseline (4.27 ± 1.3) to mid-treatment (3.24 ± 0.9 at T4), followed by a slight increase at T5 (3.60 ± 1.4), suggesting partial and non-linear improvement. Emotional rigidity demonstrated the most pronounced and sustained change, declining from 4.95 ± 1.1 at baseline to 2.62 ± 1.6 at T5, consistent with a marked increase in emotional flexibility. In contrast, behavioral rigidity remained relatively stable across follow-up, with baseline values of 3.89 ± 1.2 showing minimal variation over time (approximately 3.7). Overall, these findings indicate domain-specific changes in resistance to change, with emotional flexibility emerging as the most responsive dimension during treatment.

Dissociative symptoms were low at baseline (0.17 ± 0.13) and further decreased over time, reaching 0.08 ± 0.08 at T5, indicating that dissociative effects were mild, transient, and diminished with continued treatment (**Figure 9**).

## 4 Discussion

The present findings align with and extend a growing body of literature advocating for a shift from a purely symptom-centered model of treatment response toward a more personalized, mechanism- informed understanding of recovery in TRD. Emerging evidence suggests that improvements in psychological pain, cognitive flexibility, and higher-order reflective capacities may represent clinically meaningful processes that precede or accompany symptomatic remission, rather than mere epiphenomena of mood improvement. Within this perspective, our results indicate that esketamine’s therapeutic effects may be at least partly mediated by its impact on transdiagnostic psychological dimensions.

In particular, the marked reduction in psychache observed in our cohort supports contemporary models conceptualizing suicidal ideation and persistent distress as driven by intolerable subjective suffering rather than by depressive symptom severity alone (30). Moreover, the observed reductions in cognitive and emotional rigidity provide further support for the hypothesis that esketamine promotes psychological flexibility through neuroplastic mechanisms. Cognitive rigidity—characterized by perseverative negative thinking and impaired adaptive control—and emotional rigidity—marked by restricted affective responsiveness—are increasingly recognized as core features of treatment resistance across mood disorders. Their improvement over time in the present study suggests that esketamine may facilitate a loosening of maladaptive cognitive–emotional patterns, creating a window of opportunity for psychological reorganization and engagement in psychotherapeutic processes.

In the present study, a progressive improvement in clinical outcomes was observed over a six-month course of intranasal esketamine treatment, with remission rates showing a consistent upward trajectory across follow-up assessments. With regard to depressive symptomatology, our results confirm esketamine’s potential to induce both rapid and sustained antidepressant effects, in line with previous evidence (11, 13, 20, 31). Beyond symptom improvement, esketamine administration was associated with significant reductions in suicidal ideation and psychological distress, as reflected by both C-SSRS and PSA scores. However, the high prevalence of comorbid personality disorders in our cohort suggests that residual psychological suffering may persist despite symptomatic improvement, underscoring the need for integrative therapeutic strategies in which pharmacological stabilization facilitates engagement in psychotherapeutic interventions aimed at addressing enduring maladaptive emotional and cognitive patterns.

Beyond its well-established rapid effects on depressive symptoms and suicidal ideation (11, 13, 20, 31), our findings further suggest that esketamine modulates several key psychological dimensions— including mentalization deficits, cognitive and emotional rigidity, psychache, and social cognition— that are increasingly recognized as central to the persistence of depressive pathology and suicidal risk (32–44). In this context, the progressive increase in RMET scores observed over time indicates enhanced emotion recognition and social inference abilities, in line with prior literature describing depressive patients’ biases toward negative affective cues and difficulties in decoding positive emotional expressions (33–35).

Similarly, the progressive enhancement in reflective functioning (RFQ-8 scores), although variability acrosstime points, highlights incremental gains in mentalization abilities. As impaired mentalization is a well- acknowledged vulnerability factor in both depression and personality disorders (33), even minor improvements may yield significant benefits in emotional regulation and resilience. These findings emphasize the importance of evaluating reflective functioning as a dynamic target within the management of TRD A particularly innovative finding of this study concerns the reduction in cognitive and emotional rigidity, a core transdiagnostic dimensions across mood disorders. Cognitive rigidity, characterized by persistent negative rumination and impairments in executive functioning, and emotional rigidity, marked by a limited affective range and amplified sensitivity to negative stimuli, and that contributes to feelings of hopelessness and social isolation, are both well-documented correlates of major depressive disorder. Their observed reduction supports the hypothesis that esketamine enanhces cognitive and affective flexibility through neuroplastic mechanisms, thereby promoting adaptive coping and resilience (36–44). This interpretation aligns with contemporary models of depression that underscore deficits in flexibility, both emotional and cognitive, as core dimensions of the disorder.

The enhancement of mentalization observed in our study may, in part, mirror the overall reduction of depressive symptoms, a phenomenon that has been documented across various treatment approaches, such as SSRIs, SNRIs, and ECT (2, 4, 7, 37). Nevertheless, certain features suggest that esketamine exerts specific effects beyond those of conventional monoaminergic antidepressants. Converging lines of evidence indicate that esketamine’s modulation of glutamatergic neurotransmission via NMDA receptor antagonism leads to increased synaptic plasticity and BDNF release (14, 15, 45, 46). These neurobiological mechanisms are critically implicated not only in mood improvement but also in higher cognitive and social functions, including mentalization. Moreover, unlike SSRIs and SNRIs, which typically requiree several weeks to produce measurable changes in cognitive-emotional processing, esketamine produces rapid symptomatic relief within hours to days, potentially allowing earlier re-engagement with mentalization and relational processes, otherwise hindered during extended depressive episodes.

Preliminary evidence further supportt that esketamine enhances cognitive flexibility and reduces maladaptive rumination, creating a “window of opportunity” for psychological reorganization and integration (14, 37). The transient dissociative experience induced by esketamine may, in some patients, facilitate metacognitive distancing from distressing cognitions, indirectly supporting reflective functioning (8, 14, 18, 19).

Particularly noteworthy is the observed reduction in psychache, a deeply aversive emotional experience strongly linked suicidality. Consistent with Shneidman’s model, which conceptualizes suicide as the consequence of intolerable psychological pain rather than mental illness per se (20, 32), our results suggest that esketamine may alleviate this core affective suffering, thus reducing suicidal ideation through a distinct therapeutic mechanism. From a neurobiological perspective, this effect likely reflects enhanced neuroplasticity mediated by NMDA receptor antagonism and downstream modulation of mTOR and BDNF pathways. This plasticity may facilitate the reconnection of disrupted emotional and cognitive networks, enabling therapeutic engagement and behavioral change. Furthermore, esketamine’s modulation of limbic-prefrontal circuits may underpin its effects on emotional regulation and cognitive control (14, 15, 37, 45, 46).

**Figure 10:**
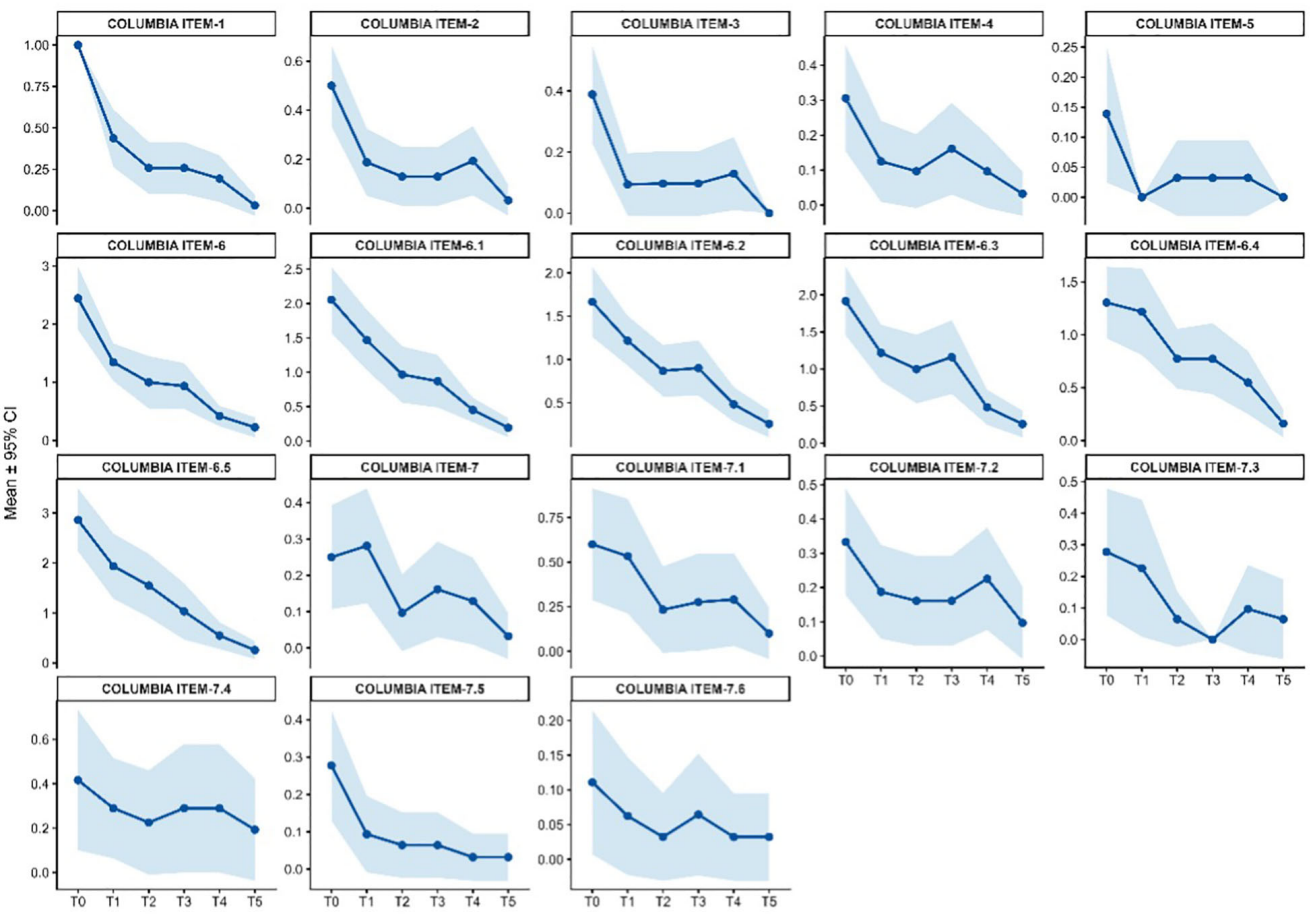
C-SSRS single-item score changes across timepoints.

**Figure 11:**
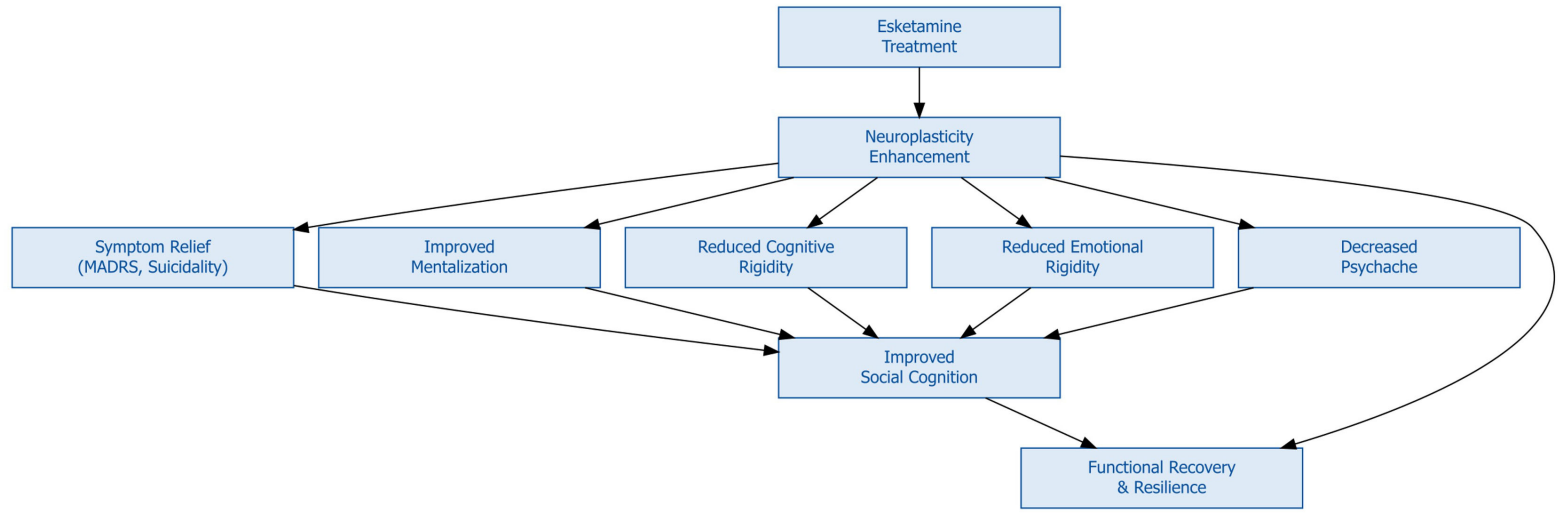
Conceptual summary of esketamine’s therapeutic pathway in Treatment- Resistant Depression (TRD).

To illustrate this complex interplay of mechanisms, **Figure 10** presents a conceptual model of esketamine’s therapeutic trajectory beginning with neuroplastic changes that lead to rapid symptom relief, followed by progressive improvements in mentalization, cognitive and emotional flexibility, and reductions in psychache. These processes collectively enhanced social cognition, allowing individuals to re-engage with social environments in more accurate and emotionally attuned ways. Ultimately, these psychological and symptomatic improvements converge toward functional recovery and the resilience (see **Figure 11**). This integrative model supports a paradigm shift toward a personalized approach to TRD and TRD-B wherein pharmacological innovation is complemented by psychological and neurocognitive interventions (8, 10, 14, 31). Rather than focusing solely on symptom suppression, treatment strategies should aim to restore psychological flexibility, reflective functioning, and emotional connectedness—core dimensions of human functioning that are often disrupted in severe depression (37, 40, 44).

## 5 Study limitations and future perspectives

Our findings should be interpreted in light of several limitations. Firs, the limited sample size may have reduced the statistical power of the analyses. To address this issue, we applied non-parametric methods (Wilcoxon signed-rank test and McNemar’s test), which are less sensitive to non-normal distribution, and used the Benjamini–Hochberg procedure to control for multiple comparison. These methodological selections were made to enhance robustness, despite the restricted cohort size. Second, the inclusion of a wide range of psychometric measures and single-item analyses provide a comprehensive characterization of esketamine’s effects but concurrently increased the likehood of type I errors. To ensure interpretative reliability, we focused primarily on findings that remained significant after the correction for multiple testing, thereby prioritizing the most dependable findings. Third, missing data were managed through complete-case analysis. Although the overall incidence of missing data was minimal and randomly distributed, this approach may have slightly reduced the effective sample size for some analyses. In addition, exploratory correlations between baseline characteristics and treatment outcomes should be interpreted with caution. These analyses were designed to generate hypotheses for prospective investigations rather than to yield definitive evidence, given the limited statistical power and potential confounders involved. The naturalistic, observational design of the study may limit the generalizability of the results. The absence of a control group, such as a placebo or active comparator, precludes definitive attribution of the observed effects to esketamine rather than to non-specific contextual or psychological factors. Moreover, the repeated administration of psychometric measures at multiple time points may have introduced test-retest effects, potentially affecting participants’ responses over time. Finally, recruitment from specialized tertiary psychiatric centers may introduce selection bias, thereby limiting the external validity of the findings to broader TRD- TRD-B populations, including those managed in primary care or community mental health settings. Another consideration is the presence of two participants with a past diagnosis of bipolar disorder. However, given their predominantly depressive clinical trajectory over the past decade, the absence of manic or hypomanic episodes, and their similarity to the rest of the sample, their inclusion is unlikely to have materially affected the overall results or their interpretation.

Future investigations should therefore aim to include larger, more heterogeneous, multisite, and demographically different sample. This approach would facilitate more robust modeling of treatment trajectories and the identification of reliable clinical and sociodemographic predictors of esketamine response. Randomized controlled trials integrating both biological and psychological outcome are essential to confirm and extend the current findings and to further elucidate the multidimensional mechanisms underlying esketamine’s therapeutic effects. Addressing these limitations will ultimately enhance the comprehension of esketamine’s efficacy and safety, contributing to the development of more personalized treatment strategies for patients with resistant depressive episode.

## 6 Conclusion

This study provides a novel and integrative contribution on esketamine’s antidepressant effects in patients with TRD and TRD-B, The observed reductions in cognitive and emotional rigidity, together with improvements in psychache, support the hypothesis that esketamine enhances adaptive coping and resilience through neuroplastic mechanisms. In summary, esketamine may act not only as a rapid antidepressant but also as a catalyst for deeper psychological change, enabling patients to move beyond symptomatic improvement toward a more sustainable and meaningful recovery. Future investigations should further explore the interplay between esketamine’s neurobiological, cognitive, and affective effects, in order to optimize its integration within comprehensive, recovery-oriented treatment strategies for TRD and TRD-B.

## Data Availability

The datasets presented in this article are not publicly available. The raw data supporting the conclusions of this study may be made available by the authors upon reasonable request. Requests to access the datasets should be directed to miriam.olivola@asst-fbf-sacco.it.

## Supplementary material

Supplementary Table 1 MADRS single-Item and total comparison over time. Wilcoxon signed-rank test; adjusted p-values reported.

Supplementary Table 2 C-SSRS single-Item comparison over time. Wilcoxon signed-rank test; adjusted p-values reported.
