# Supplementary material for: Personalizing esketamine treatment in TRD and TRBD: the role of mentalization, cognitive rigidity, psychache, and suicidality
Source: Front Psychiatry. 2026 Jan 22;16:1736114. doi: 10.3389/fpsyt.2025.1736114 (PMC12872908; doi:10.3389/fpsyt.2025.1736114)
Supplement: Supplementary Table 1 — MADRS single-Item and total comparison over time. Wilcoxon signed-rank test; adjusted p-values reported. [file Presentation1.pdf]

Table S1. MADRS single-Item and total comparison over time

Group1	Group2	Item	Adjusted p-value
T1	T0	Item 1	$p < 0.001$
T1	T0	Item 10	$p < 0.001$
T1	T0	Item 2	$p < 0.01$
T1	T0	Item 3	$p < 0.01$
T1	T0	Item 6	$p < 0.001$
T1	T0	Item 7	$p < 0.001$
T1	T0	Item 8	$p < 0.001$
T1	T0	Item 9	$p < 0.01$
T1	T0	Total	$p < 0.001$
T2	T0	Item 1	$p < 0.001$
T2	T0	Item 10	$p < 0.001$
T2	T0	Item 2	$p < 0.001$
T2	T0	Item 3	$p < 0.001$
T2	T0	Item 6	$p < 0.001$
T2	T0	Item 7	$p < 0.001$
T2	T0	Item 8	$p < 0.001$
T2	T0	Item 9	$p < 0.001$
T2	T0	Total	$p < 0.001$
T3	T0	Item 1	$p < 0.001$
T3	T0	Item 10	$p < 0.001$
T3	T0	Item 2	$p < 0.001$
T3	T0	Item 3	$p < 0.001$
T3	T0	Item 5	$p < 0.05$
T3	T0	Item 6	$p < 0.001$
T3	T0	Item 7	$p < 0.001$

Group1	Group2	Item	Adjusted p-value
T3	T0	Item 8	$p < 0.001$
T3	T0	Item 9	$p < 0.001$
T3	T0	Total	$p < 0.001$
T4	T0	Item 1	$p < 0.001$
T4	T0	Item 10	$p < 0.001$
T4	T0	Item 2	$p < 0.001$
T4	T0	Item 3	$p < 0.001$
T4	T0	Item 5	$p < 0.05$
T4	T0	Item 6	$p < 0.001$
T4	T0	Item 7	$p < 0.001$
T4	T0	Item 8	$p < 0.001$
T4	T0	Item 9	$p < 0.001$
T4	T0	Total	$p < 0.001$
T5	T0	Item 1	$p < 0.001$
T5	T0	Item 10	$p < 0.001$
T5	T0	Item 2	$p < 0.001$
T5	T0	Item 3	$p < 0.001$
T5	T0	Item 4	$p < 0.05$
T5	T0	Item 5	$p < 0.05$
T5	T0	Item 6	$p < 0.001$
T5	T0	Item 7	$p < 0.001$
T5	T0	Item 8	$p < 0.001$
T5	T0	Item 9	$p < 0.001$
T5	T0	Total	$p < 0.001$

Note. Wilcoxon signed-rank test; adjusted p-values reported.

In review

Table S2. C-SSRS single-Item comparison over time

group1	group2	Item	Adjusted p-value
T1	T0	Item 1	$p < 0.001$
T1	T0	Item 2	$p < 0.01$
T1	T0	Item 3	$p < 0.05$
T1	T0	Item 6	$p < 0.01$
T1	T0	Item 6-1	$p < 0.05$
T1	T0	Item 6-3	$p < 0.05$
T1	T0	Item 6-5	$p < 0.05$
T2	T0	Item 1	$p < 0.001$
T2	T0	Item 2	$p < 0.01$
T2	T0	Item 3	$p < 0.05$
T2	T0	Item 6	$p < 0.001$
T2	T0	Item 6-1	$p < 0.01$
T2	T0	Item 6-2	$p < 0.01$
T2	T0	Item 6-3	$p < 0.01$
T2	T0	Item 6-4	$p < 0.05$
T2	T0	Item 6-5	$p < 0.01$
T3	T0	Item 1	$p < 0.001$
T3	T0	Item 2	$p < 0.05$

group1	group2	Item	Adjusted p-value
T3	T0	Item 3	$p < 0.05$
T3	T0	Item 6	$p < 0.001$
T3	T0	Item 6-1	$p < 0.01$
T3	T0	Item 6-2	$p < 0.05$
T3	T0	Item 6-3	$p < 0.05$
T3	T0	Item 6-4	$p < 0.05$
T3	T0	Item 6-5	$p < 0.01$
T4	T0	Item 1	$p < 0.001$
T4	T0	Item 6	$p < 0.001$
T4	T0	Item 6-1	$p < 0.001$
T4	T0	Item 6-2	$p < 0.001$
T4	T0	Item 6-3	$p < 0.001$
T4	T0	Item 6-4	$p < 0.05$
T4	T0	Item 6-5	$p < 0.001$
T5	T0	Item 1	$p < 0.001$
T5	T0	Item 2	$p < 0.01$
T5	T0	Item 3	$p < 0.01$
T5	T0	Item 6	$p < 0.001$
T5	T0	Item 6-1	$p < 0.001$
T5	T0	Item 6-2	$p < 0.001$
T5	T0	Item 6-3	$p < 0.001$
T5	T0	Item 6-4	$p < 0.001$
T5	T0	Item 6-5	$p < 0.001$

Note. Wilcoxon signed-rank test; adjusted p-values reported.

In review

Figure 1.JPEG

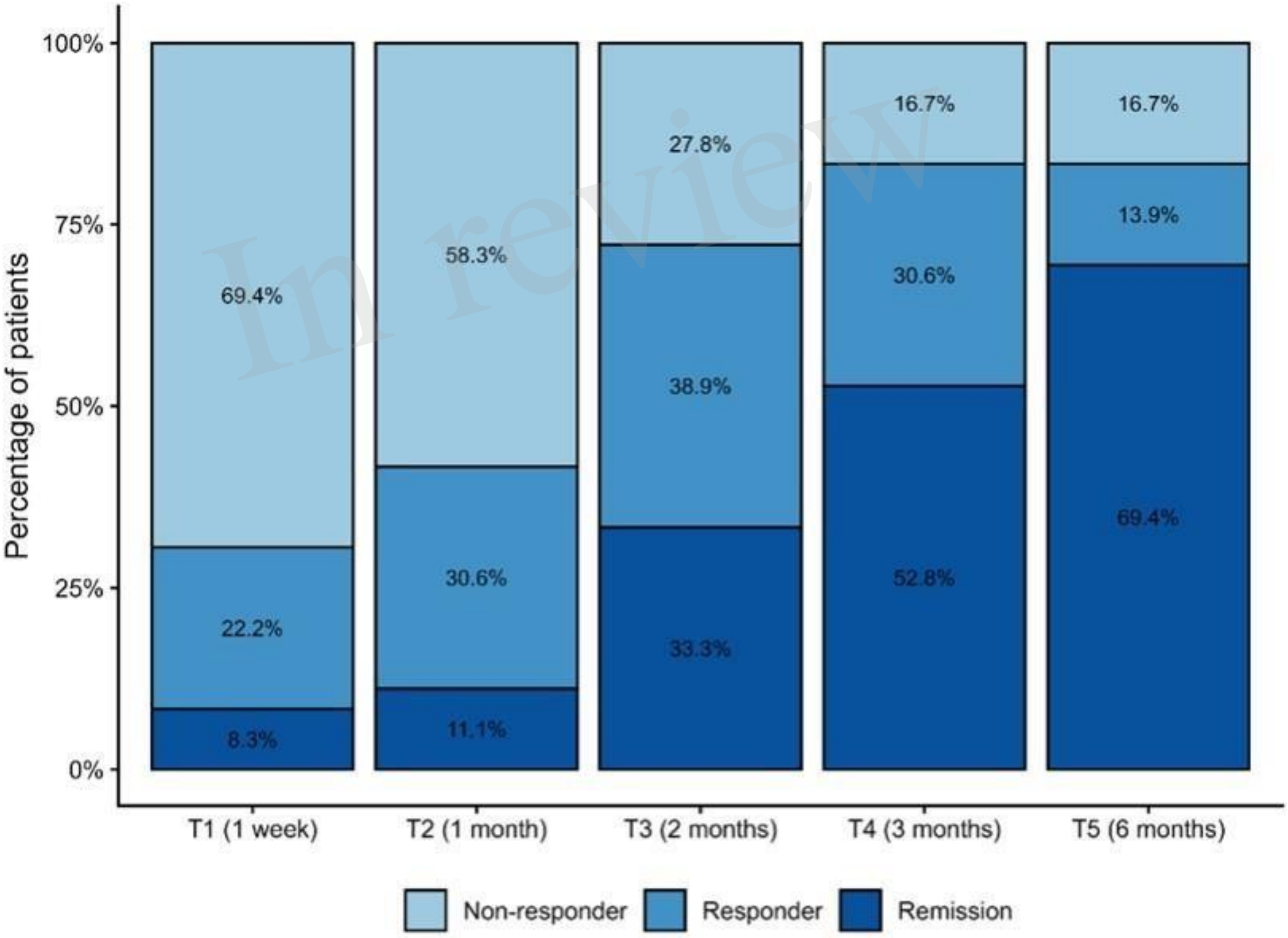

In review

Figure 2.JPEG

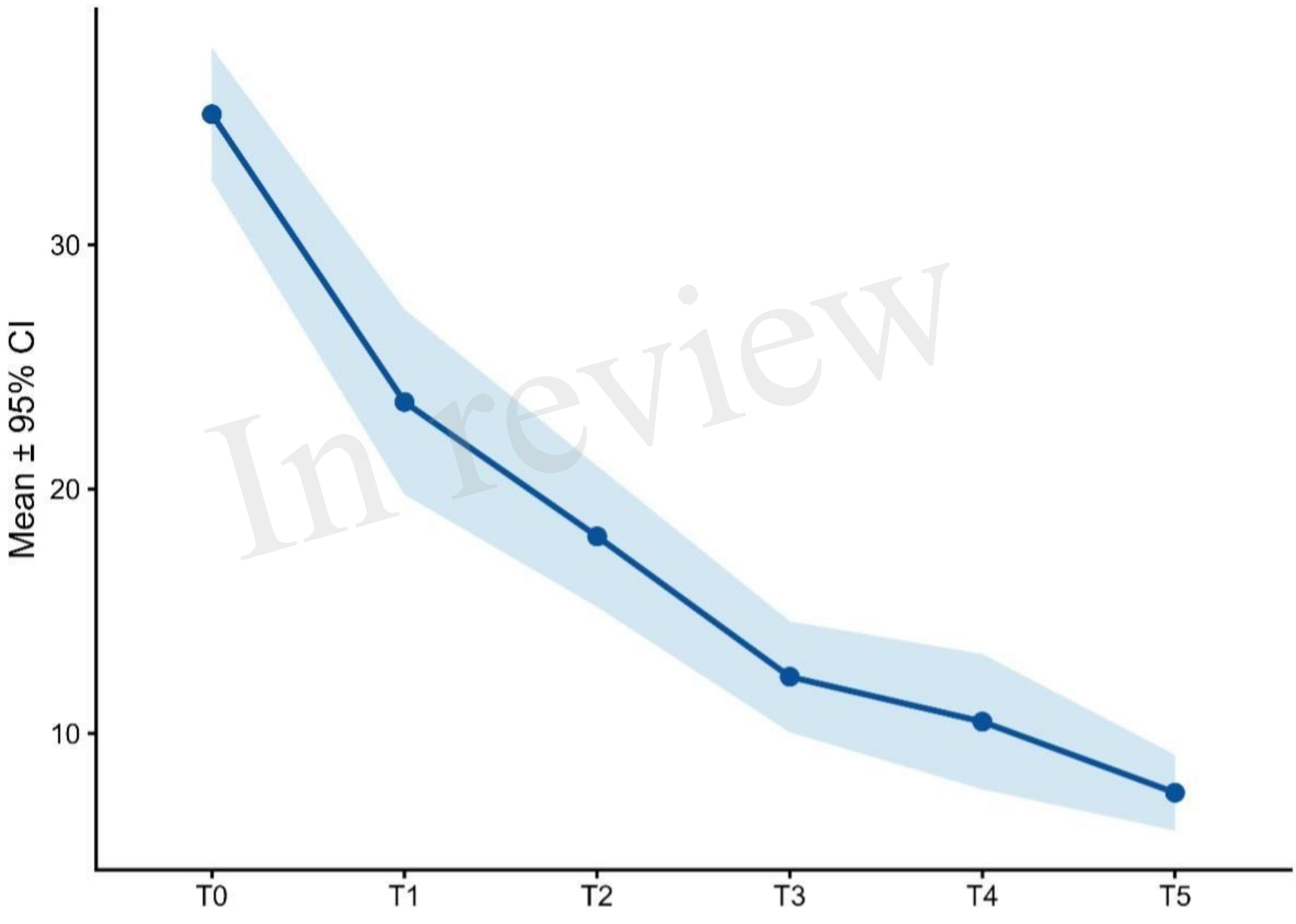

In review

Figure 3.JPEG

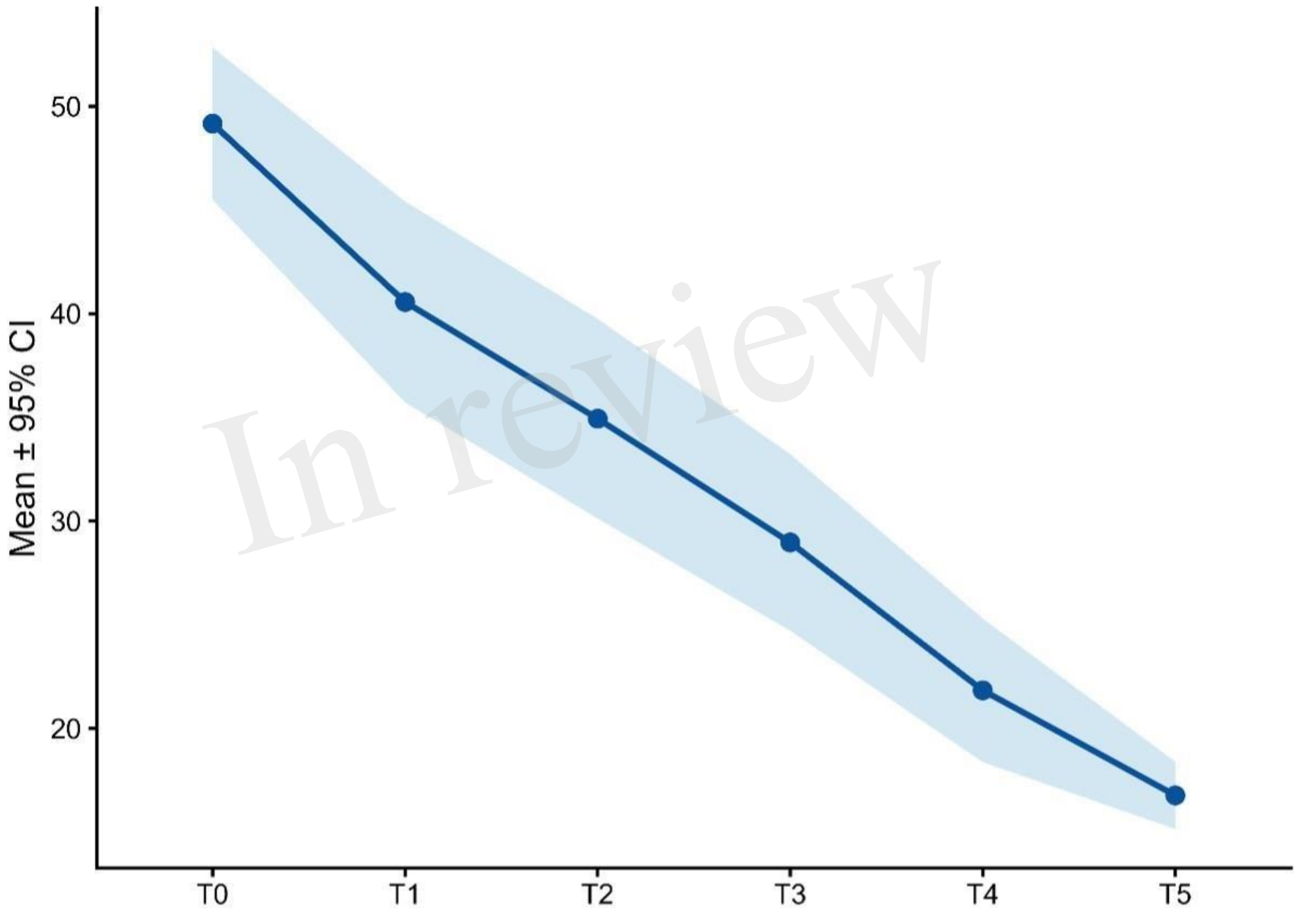

In review

Figure 4.JPEG

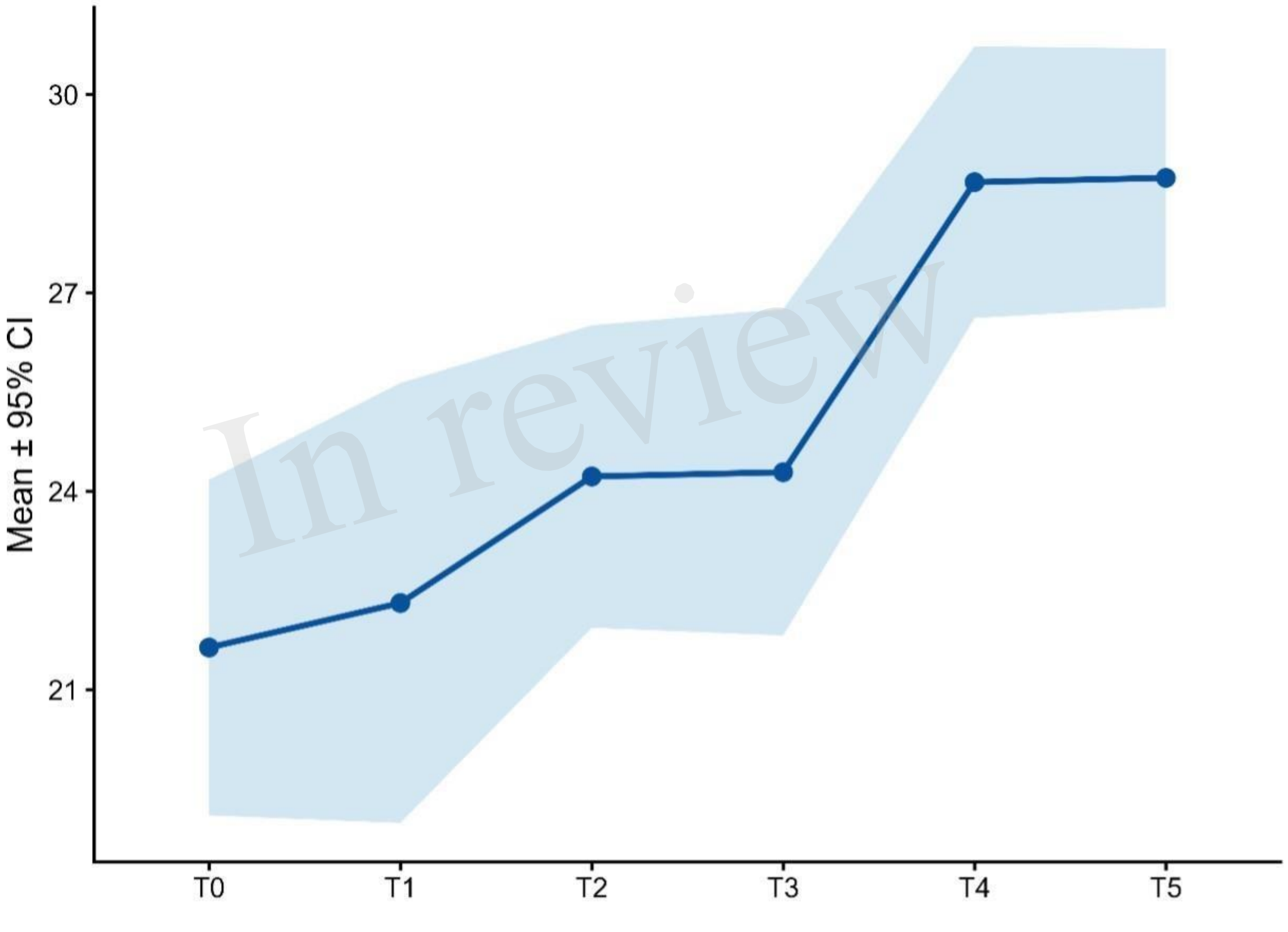

In review

Figure 5.JPEG

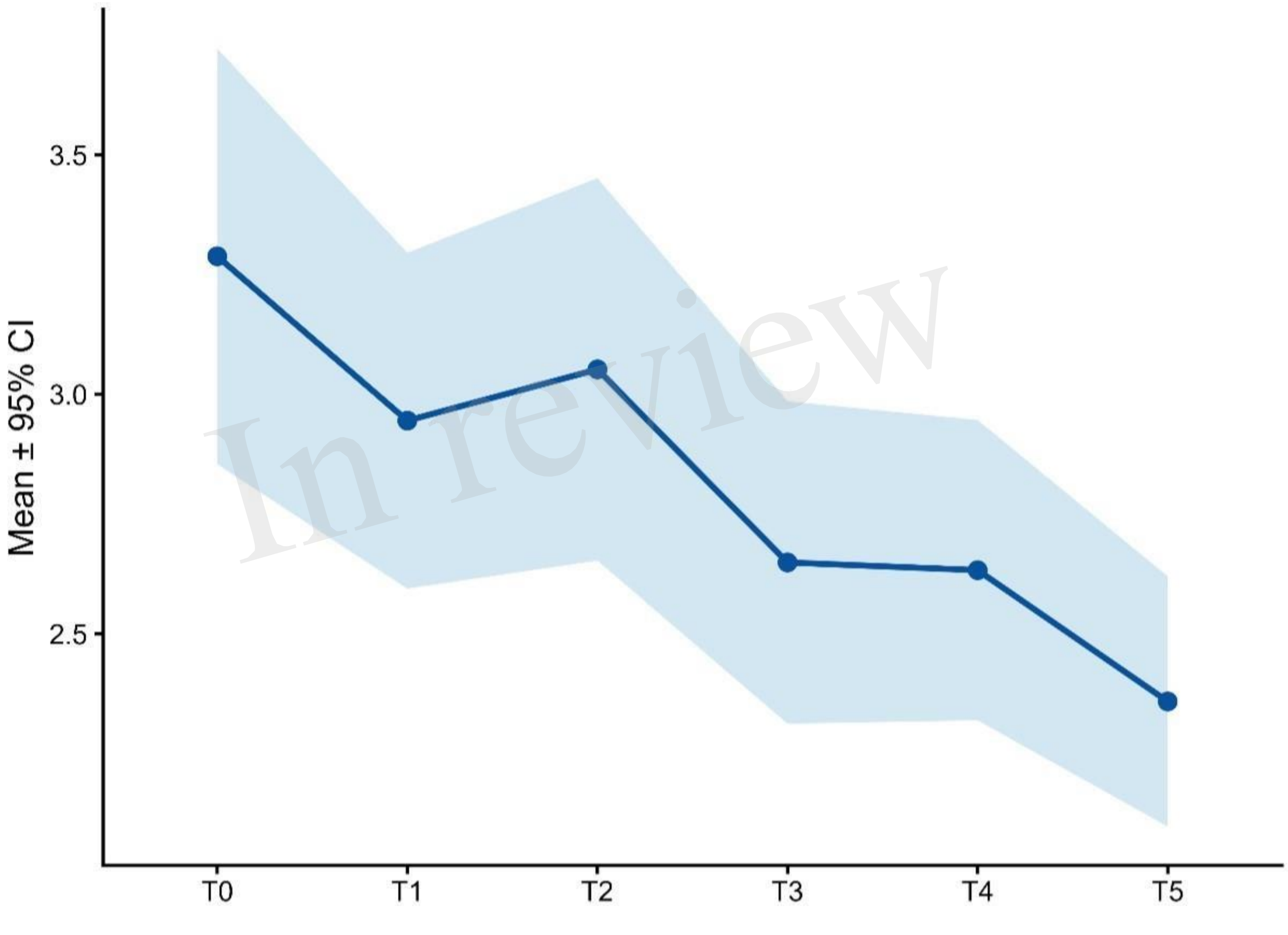

In review

Figure 6.JPEG

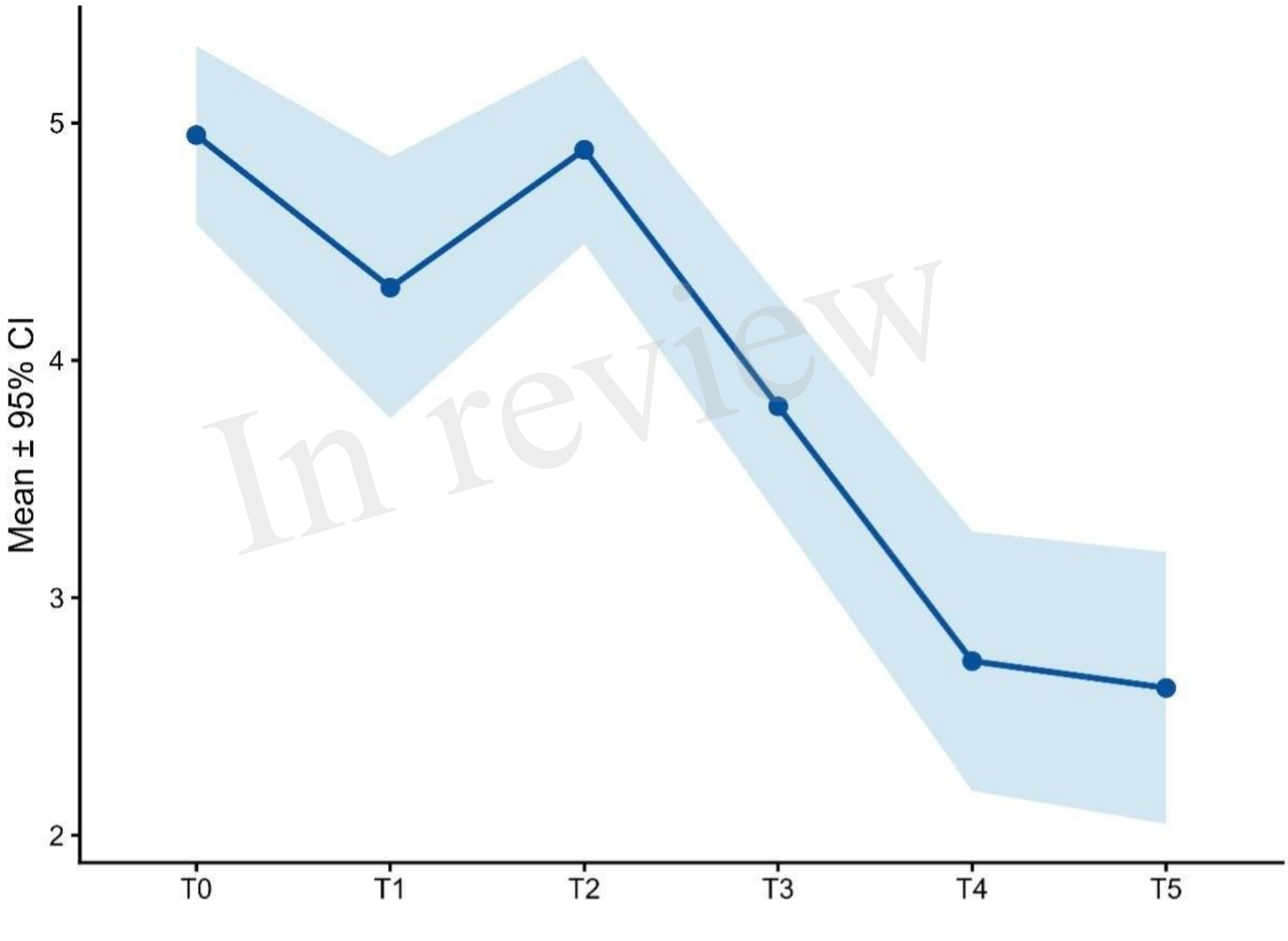

In review

Figure 7.JPEG

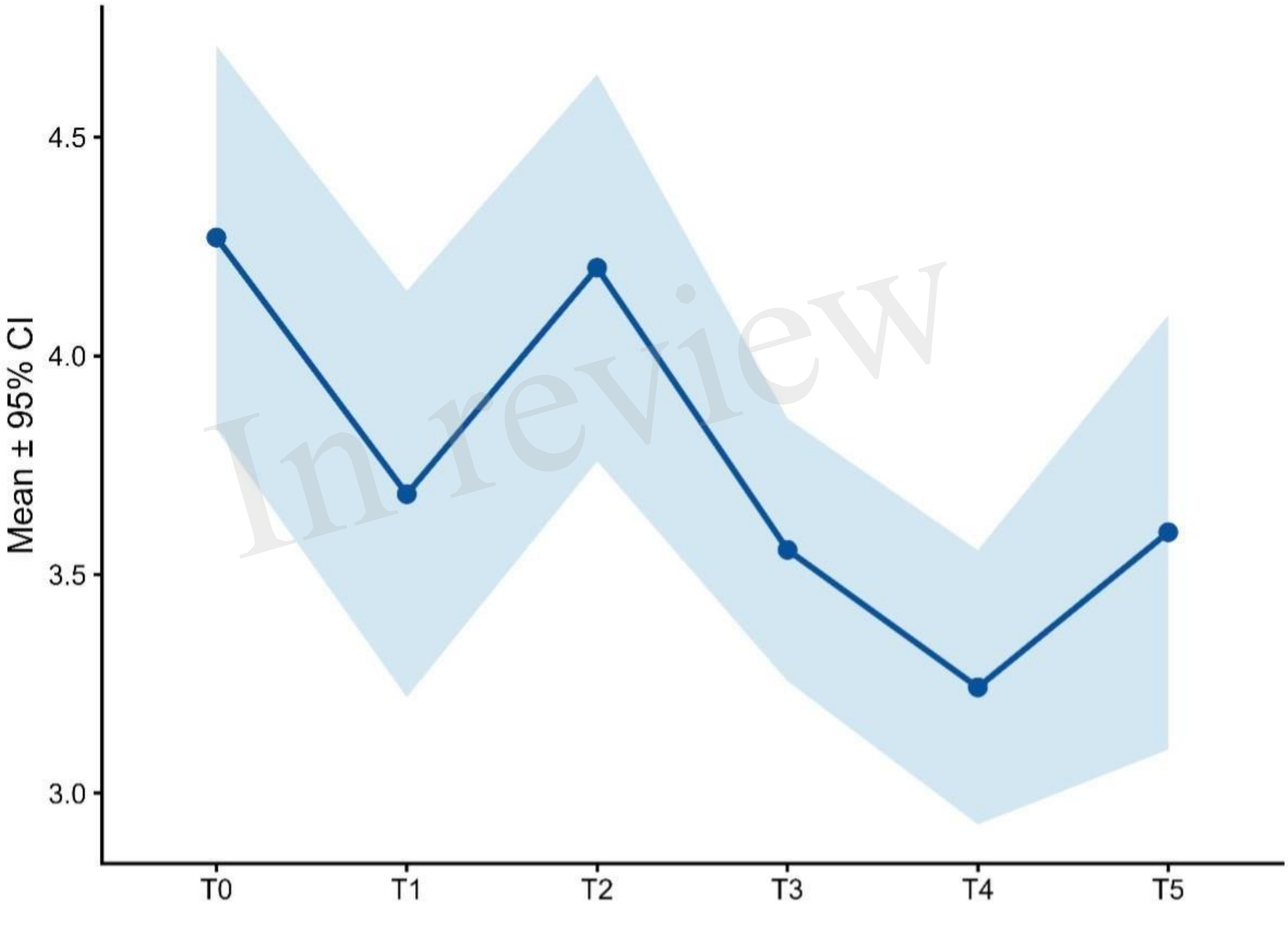

Figure 8.JPEG

In review

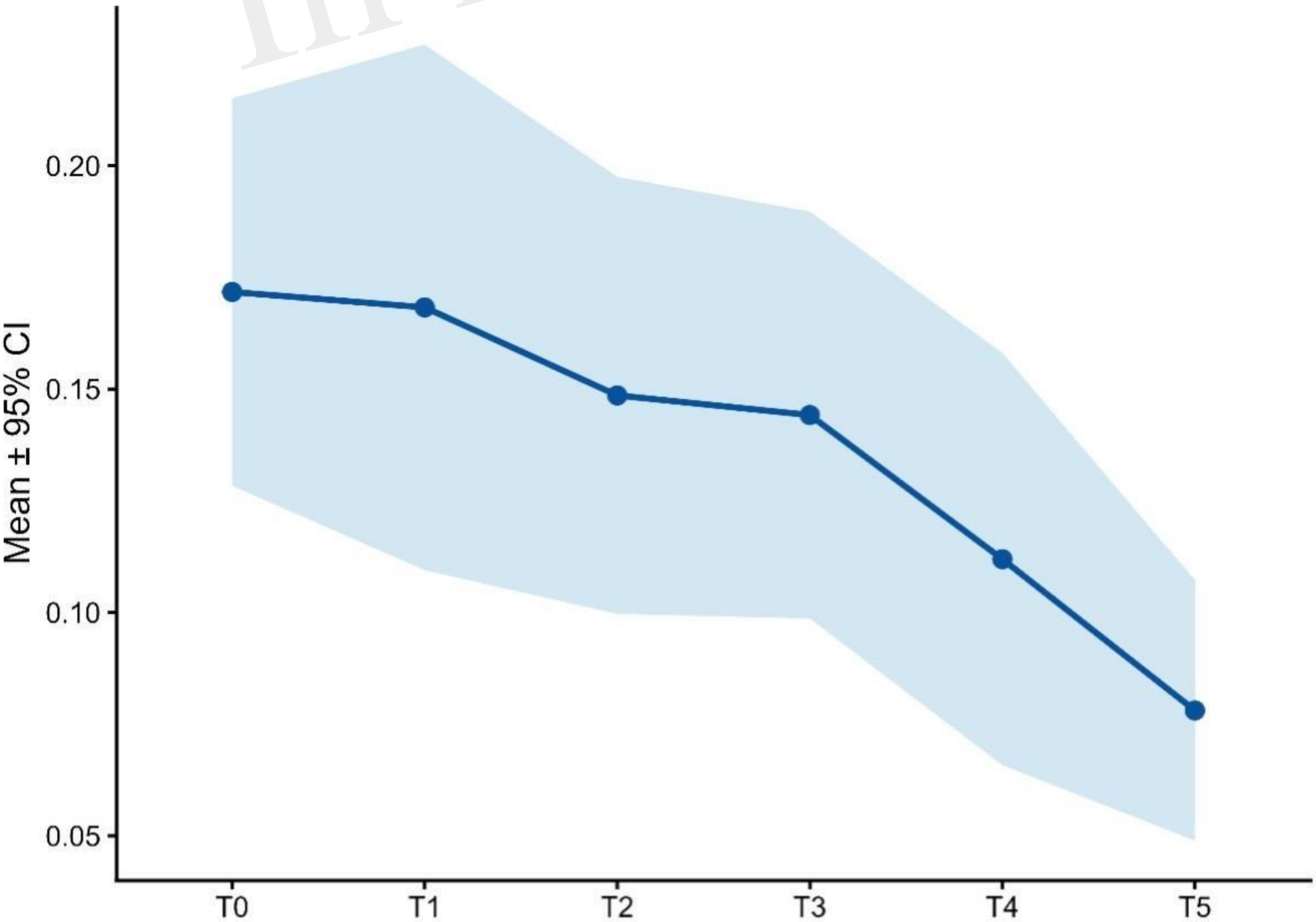

Figure 9.JPEG

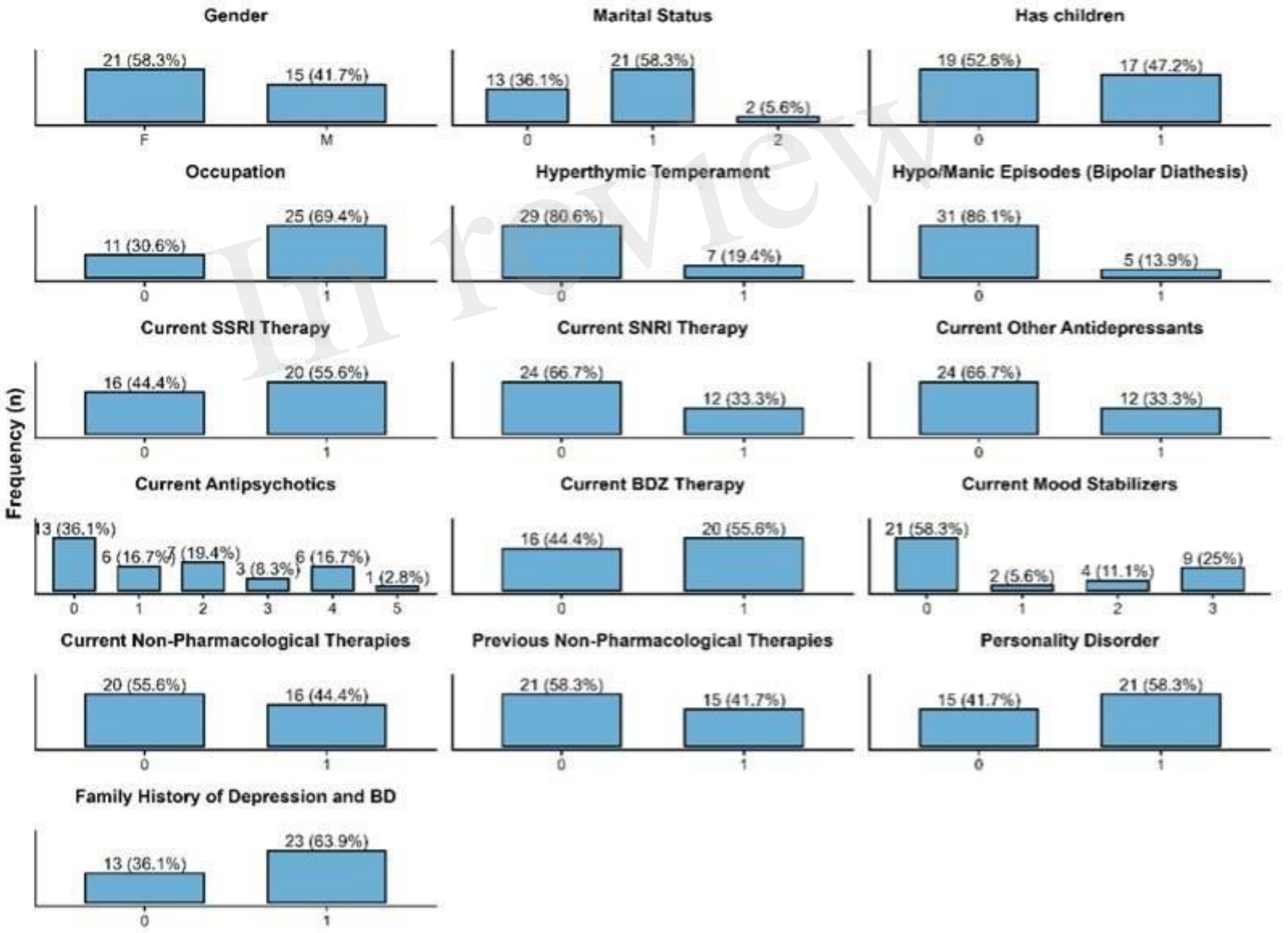

In review

Figure 10.JPEG

In review

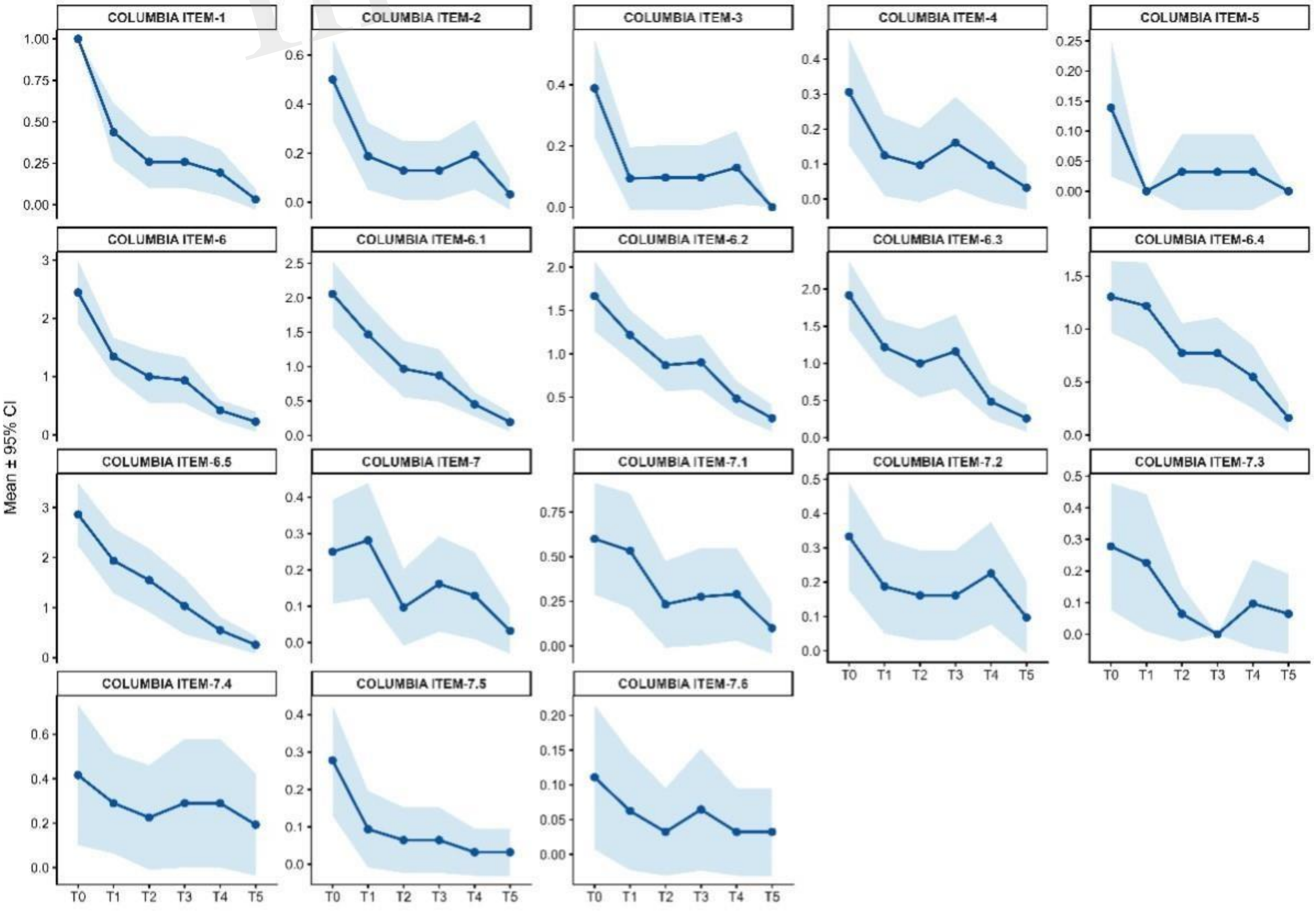

Figure 11.JPEG

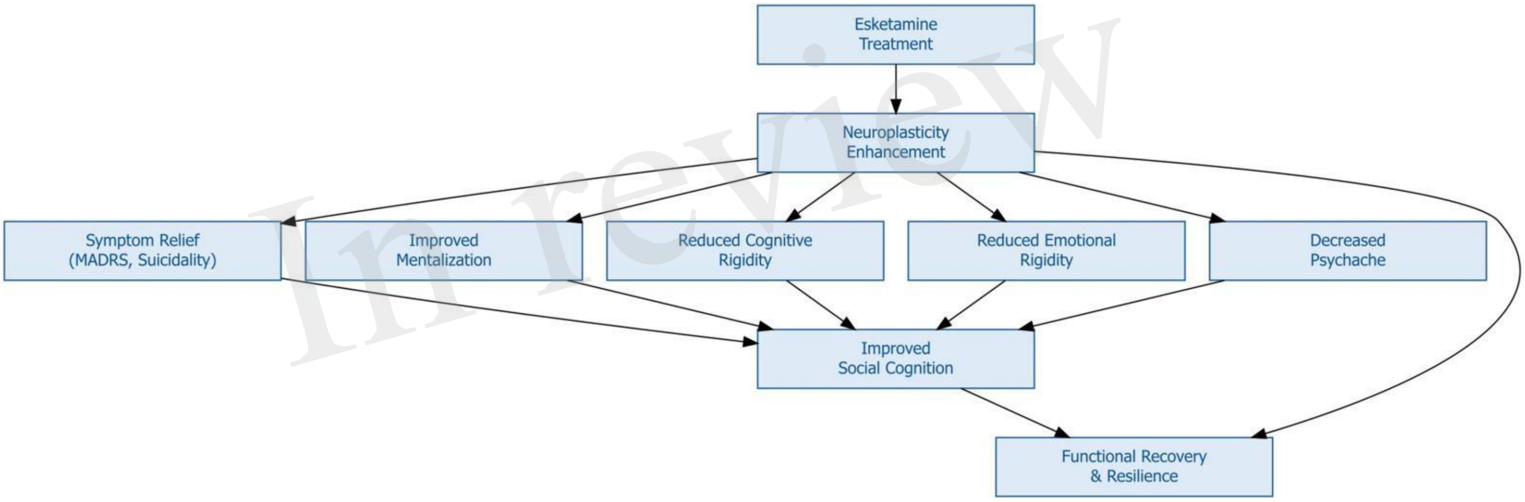

In review
